# ANGPT1–GABARAP axis modulates NLRP3 inflammasome–mediated pyroptosis in Crohn’s disease

**DOI:** 10.3389/fimmu.2026.1867825

**Published:** 2026-06-10

**Authors:** Yanchen Li, Junyan He, Ping Gao, Zeyang Fu, Yahui Guo, He Gao, Chenyang Li, Xiaolan Zhang

**Affiliations:** Department of Gastroenterology, The Second Hospital of Hebei Medical University, Hebei Key Laboratory of Gastroenterology, Hebei Institute of Gastroenterology, Hebei Clinical Research Center for Digestive Diseases, Shijiazhuang, Hebei, China

**Keywords:** ANGPT1–GABARAP axis, Crohn’s disease, Mendelian randomization, NLRP3 inflammasome, pyroptosis

## Abstract

**Background:**

Crohn**’**s disease (CD) is characterized by persistent intestinal inflammation, immune dysregulation, and intestinal barrier dysfunction. Inflammasome-mediated pyroptosis is an innate immune mechanism increasingly implicated in inflammatory bowel disease (IBD); however, the upstream molecular signals associated with NLRP3–Caspase-1–GSDMD activation in CD remain insufficiently defined. Here, we explored whether the angiopoietin-1 (ANGPT1)–gamma-aminobutyric acid receptor-associated protein (GABARAP) axis is associated with CD-related pyroptotic signaling.

**Methods:**

Protein quantitative trait locus (pQTL)-based two-sample Mendelian randomization (MR) was performed to prioritize pyroptosis-related proteins genetically associated with CD risk. Putative upstream regulators of GABARAP were then examined by two-step MR and mediation analysis. Functional validation was performed using a dextran sulfate sodium (DSS)-induced murine colitis model and LPS plus nigericin-induced cell models of NLRP3 inflammasome activation. ANGPT1–GABARAP signaling and the NLRP3–Caspase-1–GSDMD pathway were evaluated following GABARAP or ANGPT1 knockdown and exogenous recombinant human ANGPT1 (rhANGPT1) supplementation, by qRT-PCR, western blotting, ELISA, and LDH release assays.

**Results:**

MR analysis prioritized GABARAP as a suggestive protective candidate for CD, with genetically predicted higher GABARAP levels associated with a decreased disease risk (OR = 0.563, 95% CI 0.327–0.968, P = 0.038). Two-step MR further suggested a putative genetic association between ANGPT1 and GABARAP, and mediation analysis indicated that GABARAP may partially mediate the genetically predicted ANGPT1–CD association, with an estimated mediation proportion of 22.97%. DSS-induced colitis was associated with reduced ANGPT1 and GABARAP expression, along with increased NLRP3 expression, Caspase-1 processing, GSDMD-N accumulation, and elevated IL-1β, IL-18, and LDH levels. *In vitro*, silencing either GABARAP or ANGPT1 enhanced NLRP3 inflammasome-associated pyroptotic signaling under LPS plus nigericin stimulation, whereas rhANGPT1 treatment partially attenuated these responses in association with restored GABARAP expression.

**Conclusion:**

These findings support a potential role for the ANGPT1–GABARAP axis in NLRP3 inflammasome-mediated pyroptosis associated with intestinal inflammation. Together, these results provide a genetically informed framework for understanding pyroptosis-related inflammatory signaling in CD and support further investigation of the potential therapeutic relevance of this axis.

## 1 Introduction

Crohn**’**s disease (CD) is a chronic relapsing form of inflammatory bowel disease (IBD) driven by the interaction of genetic predisposition, immune dysregulation, intestinal barrier dysfunction, and environmental factors (1, 2). Although the pathobiology of CD has been increasingly clarified, the molecular basis of sustained intestinal inflammation remains incompletely understood, and effective mechanism-based therapies are still lacking.

Pyroptosis represents an inflammatory form of programmed cell death driven by inflammasome activation, Caspase-1 processing, and gasdermin D (GSDMD) cleavage (3). Accumulating evidence suggests that the activation of the NOD-like receptor family pyrin domain-containing protein 3 (NLRP3)-Caspase-1-gasdermin D (GSDMD) signaling pathway is implicated in IBD (4–8), where it may amplify intestinal inflammation through IL-1β/IL-18 release (5, 6) and contribute to intestinal barrier dysfunction (7, 8). However, the upstream molecular regulators associated with aberrant pyroptotic signaling in CD remain insufficiently characterized.

Gamma-aminobutyric acid receptor-associated protein (GABARAP), which regulates autophagy (9) and membrane trafficking (10), has not been well characterized in CD-associated pyroptotic signaling. Angiopoietin-1 (ANGPT1) participates in regulating inflammatory homeostasis and tissue integrity (11); however, its relationship with GABARAP and its relevance to CD remain unresolved. Together, these gaps suggest a potential ANGPT1–GABARAP regulatory axis associated with inflammasome-mediated pyroptotic injury.

Here, we integrated Mendelian randomization (MR) and experimental models to prioritize pyroptosis-related proteins genetically associated with CD risk, followed by two-step MR and mediation analysis to explore putative upstream regulators. The ANGPT1–GABARAP axis was further assessed in NLRP3 inflammasome–mediated pyroptotic signaling using complementary DSS-induced colitis and cell-based models of NLRP3 inflammasome activation. This integrated strategy was designed to connect genetic evidence with pyroptosis-related molecular changes in intestinal inflammation, while providing a framework for further mechanistic validation.

## 2 Materials and methods

### 2.1 Study design and summary

This study integrated protein quantitative trait locus (pQTL)-based Mendelian randomization (MR) with experimental validation to explore the relationship between the ANGPT1–GABARAP axis and NLRP3 inflammasome-associated pyroptotic signaling in intestinal inflammation. Two-sample MR was first used to prioritize circulating pyroptosis-related proteins genetically associated with CD risk; this was followed by two-step MR and mediation analysis to explore putative upstream regulators of GABARAP and estimate indirect genetic associations. Dextran sulfate sodium (DSS)-induced colitis and LPS plus nigericin-stimulated cell models of NLRP3 inflammasome activation were used for functional validation.

### 2.2 Data source and instrument selection

Pyroptosis-related proteins were first curated from the GeneCards database (http://www.genecards.org/) using “pyroptosis” as the search term. No additional GeneCards relevance-score threshold was applied; candidate genes/proteins were manually reviewed for biological relevance to inflammasome activation, gasdermin-mediated cell death, or pyroptosis-associated inflammatory signaling before being matched with available pQTL datasets. Proteins with valid genetic instruments were retained for MR analysis.

Summary-level pQTL data for pyroptosis-related proteins were obtained from the deCODE Icelandic cohort (12), whereas pQTL data for putative upstream regulators of GABARAP were obtained from the UK Biobank Pharma Proteomics Project (UKB-PPP). Summary statistics for Crohn’s disease were retrieved from the GWAS Catalog and were based on the UK Biobank fastGWA analysis (PheCode 555.1) (13).

Instrumental single nucleotide polymorphisms (SNPs) were selected at the standard genome-wide significance threshold (*P* < 5 × 10^-8^). Subsequently, linkage disequilibrium pruning was conducted using an r^2^ threshold of 0.01 across a 10-Mb region. Instrument strength was assessed using the F statistic, and weak instruments (F < 10) were excluded. Exposure and outcome datasets were harmonized before MR analysis to align effect alleles and remove ambiguous variants where appropriate.

### 2.3 Mendelian randomization analysis

A two-sample Mendelian randomization (MR) framework was utilized to assess genetically predicted associations between circulating proteins related to pyroptosis and the risk of Crohn’s disease. The summary datasets for exposure and outcome were harmonized in R (version 4.4.2) utilizing the TwoSampleMR package (14). The inverse-variance weighted (IVW) method was used as the primary analytical approach, while the weighted median and MR-Egger approaches served as complementary sensitivity analyses. To evaluate horizontal pleiotropy, instrument heterogeneity, and the reliability of the results, the MR-Egger intercept, Cochran’s Q statistic, and leave-one-out analysis were utilized, respectively. Funnel plots were also used to visually assess potential asymmetry and variant-level influence.

### 2.4 Two-step MR and mediation analysis

Proteins associated with CD risk were examined using a two-step MR and mediation framework to explore putative upstream regulators. GABARAP was specified as the mediator and CD as the outcome in the mediation model, and upstream pQTLs were treated as exposures. Total effect (β_total) was defined as the effect of pQTLs on CD; β1 denotes the effect of pQTLs on GABARAP, and β2 denotes the effect of GABARAP on CD. The mediated component was estimated as the product of β1 and β2 (β12 = β1 × β2), whereas the mediation proportion was derived by dividing β12 by the total effect and multiplying by 100%. Mediation estimates were interpreted as partial statistical indirect associations rather than definitive biological causal pathways.

### 2.5 DSS-induced colitis model

Male C57BL/6 mice maintained under specific pathogen-free conditions (6–8 weeks old, 20–23 g) were purchased from Jiangsu Huachuang Sino Pharmaceutical Technology Co., Ltd. Mice were housed in the Experimental Animal Center of the Second Hospital of Hebei Medical University, where they were maintained under controlled conditions (a 12 h-light-dark cycle, 22 ± 2 °C), and were allowed to acclimatize for 7 days. All animal procedures were approved by the Ethics Committee of the Second Hospital of Hebei Medical University (2026-AE216).

Mice were randomly assigned to control and DSS groups, with colitis induced through the administration of 2% DSS (MP Biomedicals, USA) in their drinking water for 7 days. Following this treatment, the mice received regular drinking water for 3 days (15), while the control group had access to standard drinking water ad libitum throughout the experiment. Disease activity index (DAI) scores were monitored daily during model establishment. On day 10, the mice were euthanized, and colon tissues were collected for length measurement and tissue collection.

### 2.6 Histological assessment of colitis

On day 10, mice were euthanized under 1.5% isoflurane anesthesia, followed by cervical dislocation. Colon tissues were processed for hematoxylin and eosin (H&E) staining. Histopathological damage was evaluated on the basis of inflammatory cell infiltration, mucosal injury, glandular architectural disruption, and ulceration, with each parameter graded from 0 to 4 (15). The maximum total histological score was 16, with higher scores indicating more severe histopathological damage. The remaining colon tissues were reserved for subsequent protein extraction and biochemical assays.

### 2.7 Cell culture and treatment

THP-1 cells were obtained from the Institute of Biochemistry and Cell Biology, Chinese Academy of Sciences (Shanghai, China), whereas NCM460 cells were obtained from Qisai Biotechnology Co., Ltd. (Wuhan, China). Both cell lines were cultured in RPMI-1640 medium with 10% fetal bovine serum(Gibco, USA) and 1% penicillin-streptomycin solution (SolarBio, China) at 37 °C under 5% CO_2_. THP-1 cells were treated with phorbol 12-myristate 13-acetate (PMA, 100 nM; MedChemExpress, USA) for 24 h to induce differentiation into adherent macrophage-like cells (16), while NCM460 cells were directly subjected to the subsequent experiments.

Cells were transfected with candidate siRNAs targeting GABARAP or ANGPT1, or negative control siRNA (siNC) (all from APEXBIO, USA) using Lipofectamine 2000 (Invitrogen, USA) for siRNA screening. Knockdown efficiency was assessed at 24 h after transfection by qRT-PCR and at 48 h by Western blotting. siRNAs achieving the highest knockdown efficiency were used for further experiments.

Control cells were left untreated. Following 48 h of siRNA transfection, cells were pre-incubated with rhANGPT1 (200 ng/mL; MedChemExpress, USA) for 30 min (17). This was followed by LPS (1 μg/mL; Sigma-Aldrich, USA) for 4 h, and nigericin (10 μM; MedChemExpress, USA) for 2 h (18). Cells and supernatants were harvested for analysis.

### 2.8 Reverse transcription and qRT-PCR

Total RNA was extracted from colonic tissues or cultured cells and subsequently reverse-transcribed to generate cDNA (ShareBio, China). Transcript levels of ANGPT1, GABARAP, IL1B, and IL18 were measured by quantitative real-time PCR (qRT-PCR; ShareBio, China). Knockdown efficiency of siRNA was assessed 24 h after transfection. Relative mRNA abundance was evaluated using the 2^-ΔΔCt^ method, with β-actin serving as the internal control. Primer sequences are listed in **Supplementary Table 1**.

### 2.9 Western blotting

Proteins from colon tissues and cultured cells were extracted with RIPA buffer containing phenylmethylsulfonyl fluoride (PMSF; Solarbio, China), and quantified by BCA assay (Solarbio, China). Equal amounts of protein were separated by SDS–PAGE, transferred to PVDF membranes, and incubated with primary antibodies against NLRP3 (Abcam, UK), ANGPT1 (HuaAn Biotechnology, China), GABARAP (HuaAn Biotechnology, China), GSDMD (Abiowell, China), Caspase-1 (Abcam, UK), and β-actin (Proteintech, China), followed by fluorescent secondary antibodies. Signals were detected using a near-infrared imaging system and quantified with ImageJ after normalization to β-actin. For cell-based Western blotting experiments, three independent transfection experiments were performed, and protein lysates from each experiment were collected separately. Where feasible, lysates from the three independent experiments were quantified in the same BCA assay batch and analyzed under the same Western blotting conditions to minimize technical variation. For experiments involving multiple target proteins, target proteins were detected on separate membranes using lysates prepared from the same experimental groups, and each membrane was probed with its corresponding β-actin loading control for normalization. For figure presentation, representative western blot lanes are shown, whereas densitometric quantification was performed using data from all three independent experiments. GSDMD cleavage and Caspase-1 activation were assessed using the GSDMD-N/GSDMD-FL and Caspase-1 (p10)/pro-Caspase-1 ratios, respectively (6–8).

### 2.10 ELISA and LDH release assays

Tissue homogenates were prepared in cold PBS and clarified by centrifugation. Concentrations of ANGPT1, GABARAP, IL-1β, and IL-18 in colon tissue homogenates and cell culture supernatants were measured using ELISA, with kits obtained from Kelu Biotechnology (China) for ANGPT1 and GABARAP and from Lianke Biotechnology (China) for IL-1β and IL-18. LDH activity in colon tissue homogenates was measured using an LDH assay kit from Nanjing Jiancheng Bioengineering Institute (China). Tissue measurements were normalized to total protein content.

### 2.11 Statistical analysis

All MR analyses and visualization were conducted in R (version 4.4.2) using the TwoSampleMR and RMediation packages (14). To evaluate instrument heterogeneity, Cochran’s Q test was employed, while the MR-Egger intercept was used to assess directional pleiotropy. Sensitivity analyses included weighted median analysis, MR-Egger regression, funnel plots, and leave-one-out analysis. For the MR screen of 24 pyroptosis-related proteins, Bonferroni correction was applied by setting the significance threshold at 0.05/24. Associations with *P* < 0.05 that did not meet this corrected threshold were interpreted as suggestive findings.

Experimental data from animal and cell studies were analyzed using GraphPad Prism version 10.0. Data are presented as mean ± SD, with sample sizes indicated in the corresponding figure legends. All statistical tests were two-sided. Differences between two groups were evaluated by unpaired two-tailed Student’s t test, whereas comparisons among multiple groups were analyzed by one-way ANOVA followed by Tukey’s multiple comparisons test. *P* < 0.05 was considered statistically significant unless otherwise specified.

## 3 Results

### 3.1 GABARAP is associated with reduced Crohn’s disease risk

**Figure 1:**
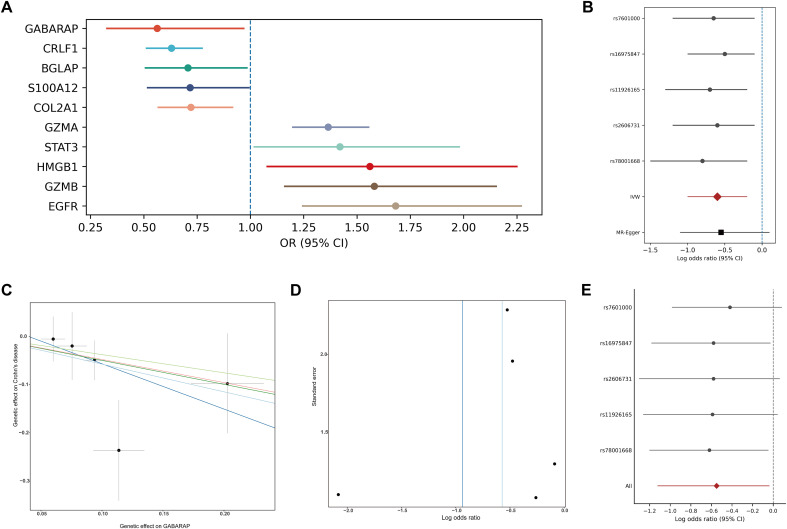
GABARAP is associated with reduced Crohn’s disease risk. **(A)** Two-sample MR analysis of pyroptosis-related proteins in relation to Crohn’s disease risk. Points denote odds ratios (ORs) and 95% confidence intervals (CIs); horizontal error bars indicate 95% CIs, and the dashed line indicates OR = 1. **(B)** Forest plot of SNP-level effect estimates for GABARAP on CD risk, with pooled inverse-variance-weighted (IVW) and MR-Egger estimates shown on the log odds ratio scale. **(C)** Scatter plot of SNP-specific effects on GABARAP (exposure) versus CD (outcome), with fitted lines showing MR estimates from different MR methods. **(D)** Funnel plot of SNP-level estimates against their standard errors; symmetry indicates no marked evidence of directional horizontal pleiotropy. **(E)** Leave-one-out analysis of the MR estimate; each point reflects exclusion of a single SNP.

Two-sample Mendelian randomization using deCODE pQTL data prioritized 24 candidate pyroptosis-related proteins, of which 23 showed nominal associations with CD risk (*P* < 0.05; **Figure 1A**; **Supplementary Table 2**). Among these candidates, GABARAP showed a consistent inverse association with CD risk and was retained as a suggestive MR candidate for downstream experimental validation. The IVW model indicated an inverse association between genetically predicted GABARAP levels and the risk of CD [odds ratio (OR) = 0.563, 95% confidence interval (CI) 0.327–0.968, *P* = 0.038; **Figure 1A**].

Instrument-specific estimates for GABARAP were consistent, with no evidence of outliers in the forest plot (**Figure 1B**). The IVW and MR-Egger estimates showed concordant effect directions in scatter plots (**Figure 1C**). Symmetry of the funnel plot, together with a non-significant MR-Egger intercept, suggested no evidence of marked horizontal pleiotropy (**Figure 1D**). Leave-one-out analysis further showed that the overall association was robust and not dependent on any single nucleotide polymorphism (SNP; **Figure 1E**), supporting the stability of the genetically predicted association between GABARAP and CD risk.

### 3.2 ANGPT1–GABARAP axis is associated with Crohn’s disease risk

**Figure 2:**
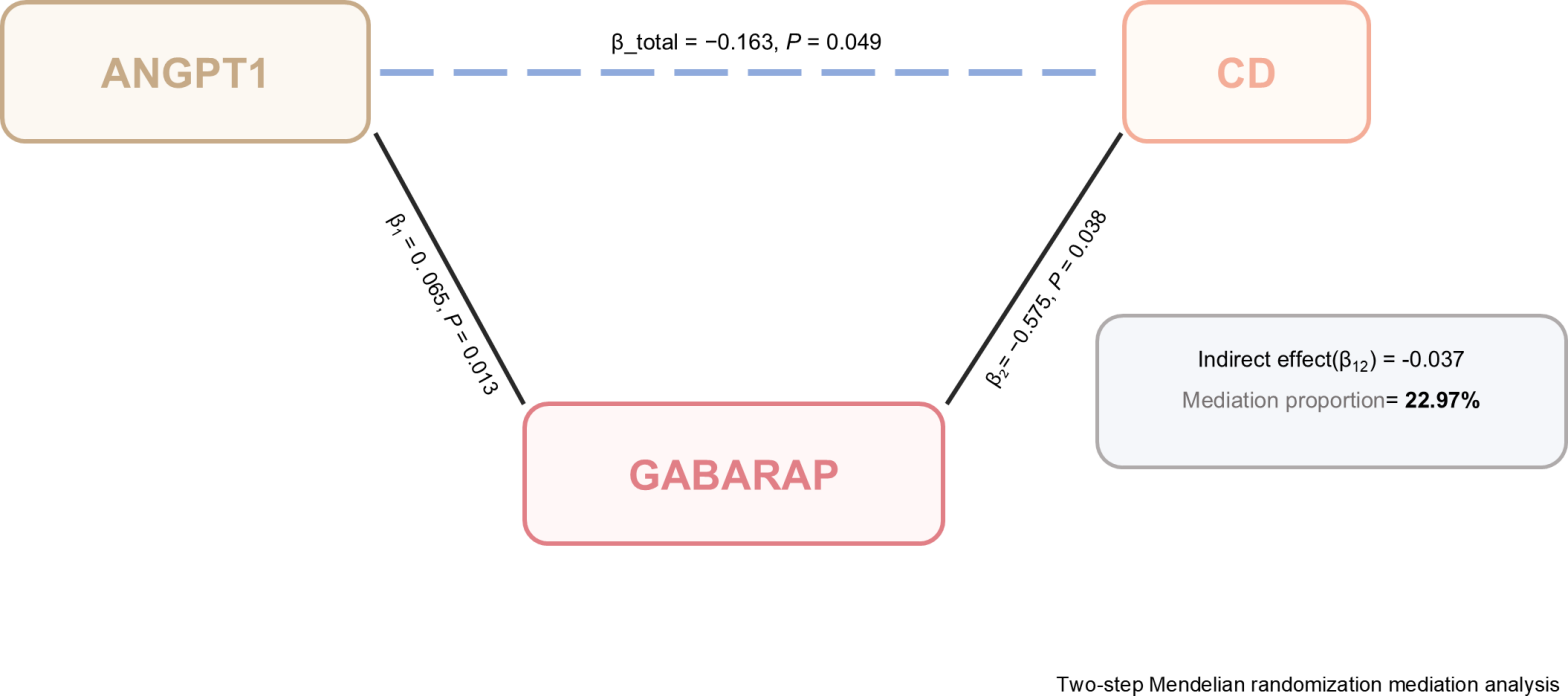
ANGPT1–GABARAP axis is associated with Crohn’s disease risk. Two-step MR analysis suggested a putative genetic relationship between ANGPT1 and GABARAP. ANGPT1 showed a positive association with GABARAP, whereas GABARAP was linked to reduced CD risk. Mediation analysis suggested a partial statistical indirect association involving GABARAP, representing 22.97% of the total effect. These estimates should be interpreted as genetically predicted associations rather than evidence of a definitive causal hierarchy.

Two-step MR suggested a putative genetic relationship between ANGPT1 and GABARAP. Genetically predicted ANGPT1 levels were positively associated with GABARAP abundance (β_1_ = 0.065, *P* = 0.013), whereas GABARAP was inversely associated with CD risk (β_2_ = −0.575, *P* = 0.038). ANGPT1 exhibited a nominal total effect on CD risk (β_total = −0.163, *P* = 0.049). Mediation analysis suggested that GABARAP may partially mediate the genetically predicted ANGPT1-CD association, with an indirect effect of β_12_ = −0.037, *P* = 0.093, representing 22.97% of the total effect. These findings support a putative ANGPT1–GABARAP relationship associated with CD risk, while indicating partial statistical mediation rather than a definitive causal hierarchy (**Figure 2**).

### 3.3 ANGPT1–GABARAP axis is associated with pyroptosis activation in colitis

**Figure 3:**
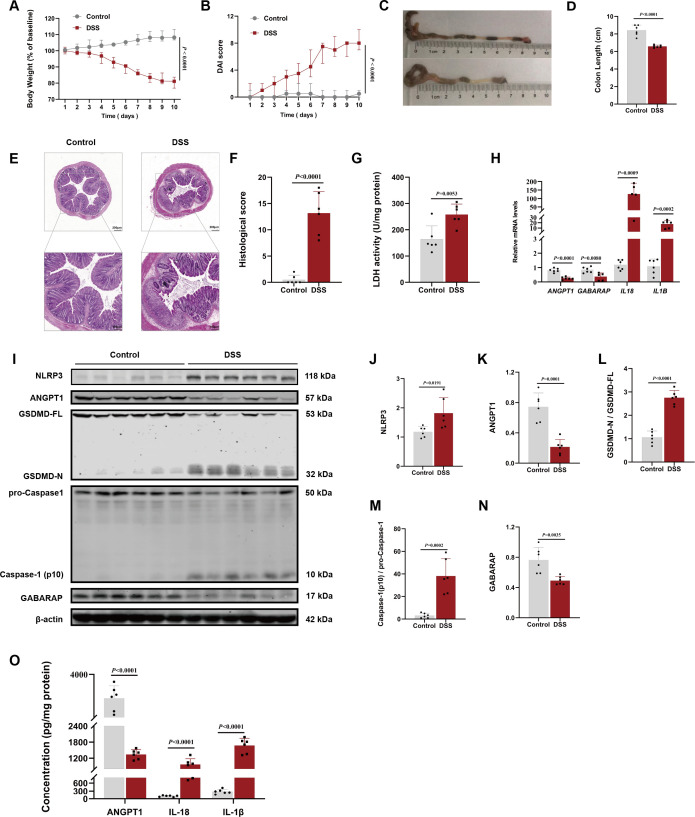
ANGPT1–GABARAP axis is associated with pyroptosis activation in DSS-induced colitis. **(A)** Body weight change (% of baseline). **(B)** Disease activity index (DAI). **(C)** Representative colon morphology. **(D)** Colon length. **(E)** Representative H&E-stained colon sections. Scale bars as indicated. **(F)** Histological scores. **(G)** LDH activity in colon tissue homogenates normalized to total protein content. **(H)** Relative mRNA expression of ANGPT1, GABARAP, IL18 and IL1B. **(I)** Western Blotting analysis of NLRP3, ANGPT1, GSDMD-FL, GSDMD-N, pro-Caspase-1, Caspase-1 (p10), GABARAP and β-actin. **(J–N)** Quantification of NLRP3 **(J)**, ANGPT1 **(K)**, GSDMD cleavage (GSDMD-N/GSDMD-FL) **(L)**, Caspase-1 activation (Caspase-1 (p10)/pro-Caspase-1) **(M)**, and GABARAP **(N)**, normalized to β-actin. **(O)** ELISA quantification of ANGPT1, IL-18, and IL-1β in colon tissue. Mice received 2% DSS in drinking water for 7 days followed by regular water for 3 days; controls received regular water throughout. Western Blotting quantification was normalized to the corresponding β-actin loading control. Data are shown as mean ± SD (n ≥ 5). Statistical significance was assessed using an unpaired two-tailed Student’s t-test.

DSS-induced colitis showed progressive body weight loss, increased DAI scores, colon shortening, and marked histological injury (**Figures 3A–F**). ANGPT1 and GABARAP expression were reduced following DSS exposure, with concurrent increases in IL-18 and IL-1β levels, as well as enhanced LDH activity normalized to total protein content (**Figures 3G, H, O**). Western Blotting showed that NLRP3 was upregulated, with enhanced Caspase-1 processing and accumulation of GSDMD-N (**Figures 3I–N**). Reduced ANGPT1 and GABARAP expression paralleled enhanced NLRP3 inflammasome-associated pyroptotic signaling in this experimental colitis model, supporting a potential association between this axis and inflammasome-related inflammatory injury.

### 3.4 GABARAP knockdown enhances NLRP3 inflammasome-associated pyroptotic signaling

**Figure 4:**
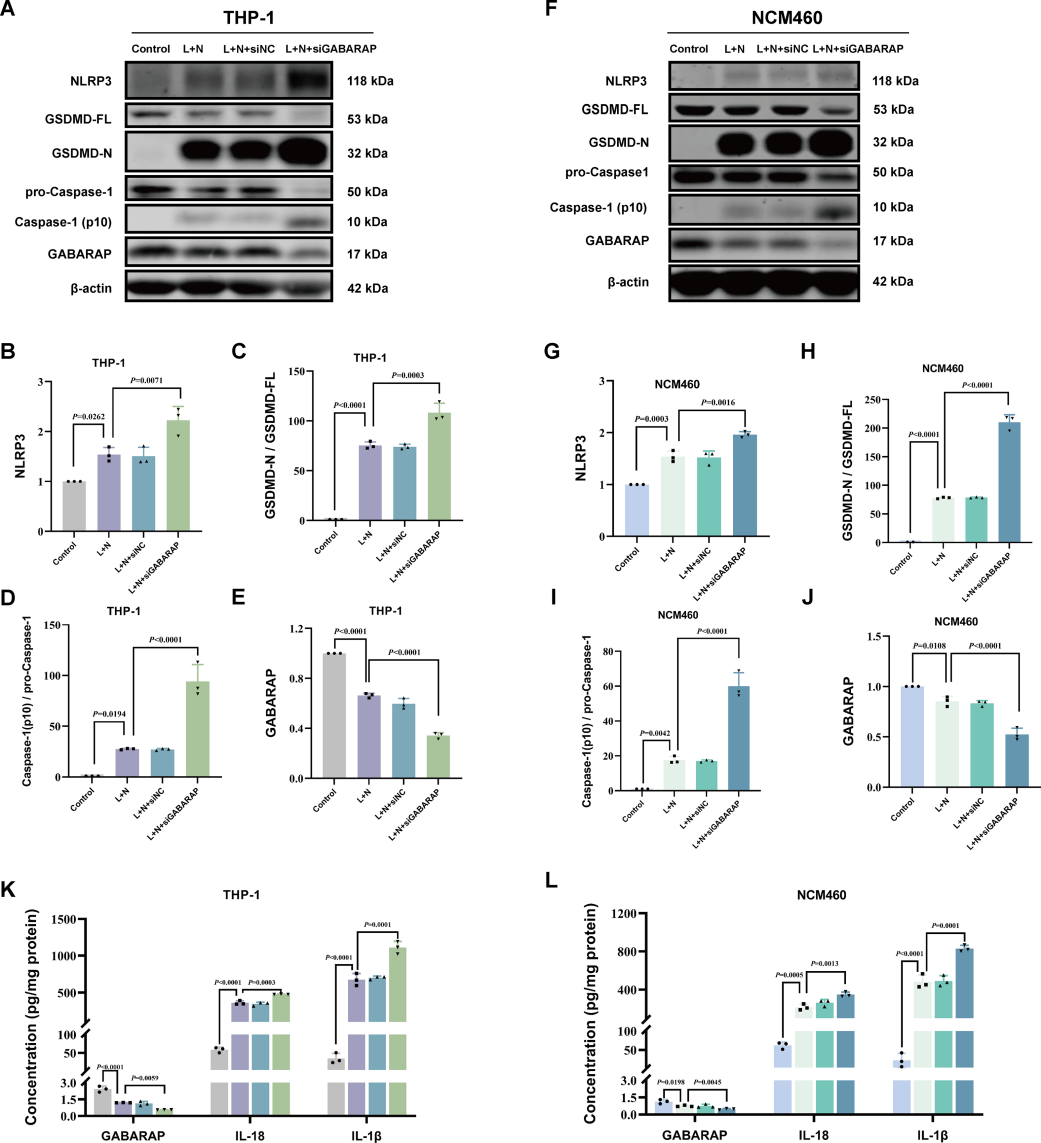
GABARAP knockdown enhances NLRP3 inflammasome-associated pyroptotic signaling in cell models. **(A, F)** Representative western blots of NLRP3, GSDMD-FL, GSDMD-N, pro-Caspase-1, Caspase-1 (p10), GABARAP, and β-actin in THP-1-derived macrophages and NCM460 cells. **(B–E, G–J)** Quantification of NLRP3 **(B, G)**, GSDMD cleavage [GSDMD-N/GSDMD-FL; **(C, H)**], Caspase-1 activation [Caspase-1 (p10)/pro-Caspase-1; **(D, I)**], and GABARAP **(E, J)**, normalized to β-actin. **(K, L)** ELISA quantification of GABARAP, IL-18, and IL-1β in culture supernatants. After 48 h of siRNA transfection, cells were stimulated with LPS (1 μg/mL, 4 h) followed by nigericin (10 μM, 2 h); unstimulated cells served as controls. Representative western blots are shown, and densitometric quantification was based on three independent transfection experiments. NLRP3, GSDMD, and Caspase-1/GABARAP were detected on separate membranes with corresponding β-actin controls. Data are shown as mean ± SD (n = 3) and analyzed by one-way ANOVA followed by Tukey’s multiple comparisons test.

THP-1-derived macrophages and NCM460 epithelial cells were stimulated by LPS plus nigericin, with efficient GABARAP knockdown confirmed (**Supplementary Figure 1**). In both cell models, LPS and nigericin stimulation induced NLRP3 inflammasome-associated pyroptotic signaling, as reflected by increased NLRP3 expression, higher levels of Caspase-1 (p10) and GSDMD-N (**Figures 4A–J**), and enhanced secretion of IL-1β and IL-18 (**Figures 4K, L**). GABARAP knockdown further enhanced these pyroptosis-associated molecular changes (**Figures 4A–L**), supporting an association between GABARAP expression and NLRP3–Caspase-1–GSDMD signaling under LPS plus nigericin stimulation.

### 3.5 ANGPT1 attenuates pyroptotic signaling accompanied by GABARAP restoration

**Figure 5:**
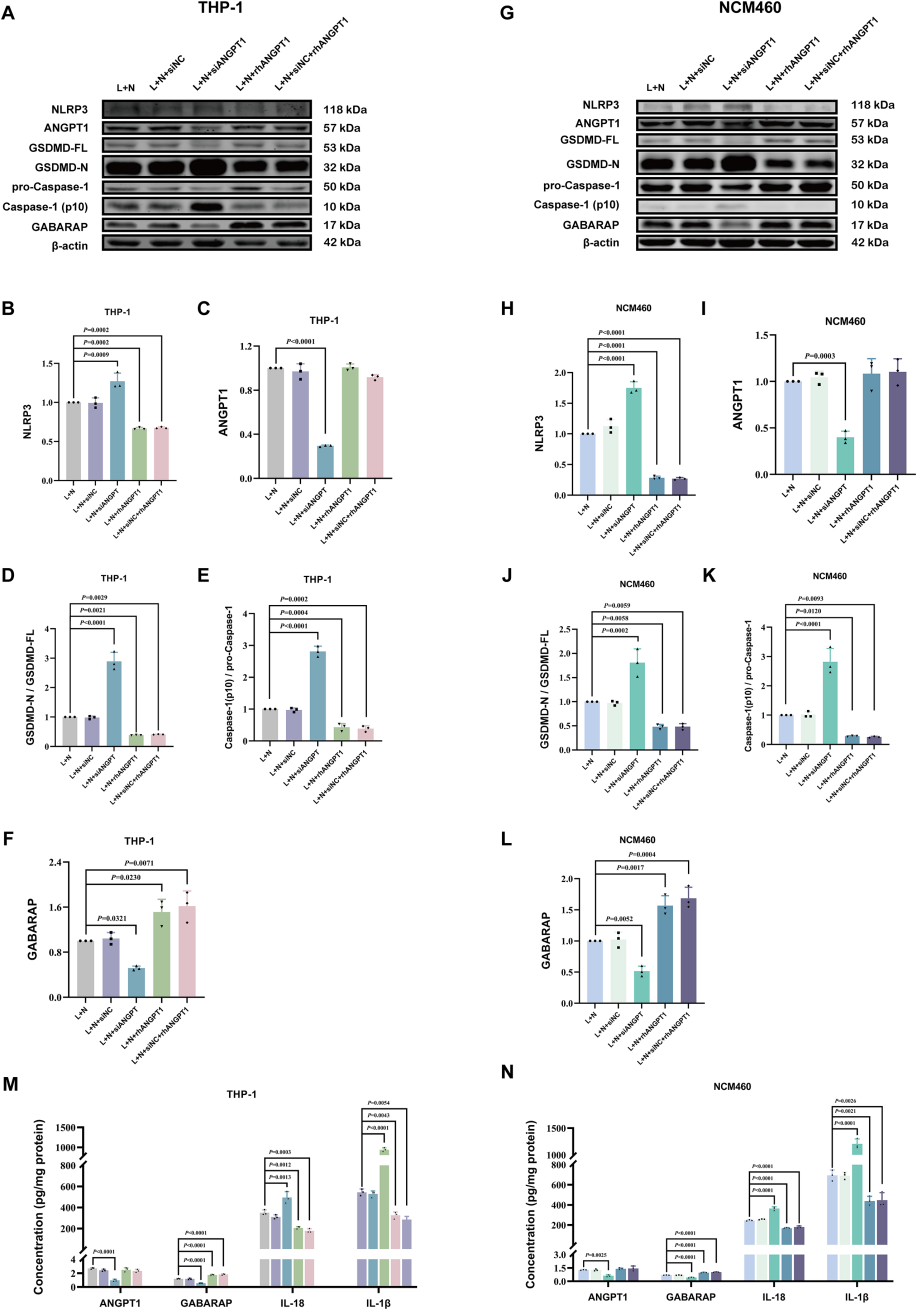
ANGPT1 attenuates pyroptotic signaling accompanied by GABARAP restoration. **(A, G)** Representative western blots of NLRP3, ANGPT1, GSDMD-FL, GSDMD-N, pro-Caspase-1, Caspase-1 (p10), GABARAP, and β-actin in THP-1-derived macrophages and NCM460 cells. **(B–F, H–L)** Quantification of NLRP3 **(B, H)**, ANGPT1 **(C, I)**, GSDMD cleavage [GSDMD-N/GSDMD-FL; **(D, J)**], Caspase-1 activation [Caspase-1 (p10)/pro-Caspase-1; **(E, K)**], and GABARAP **(F, L)**, normalized to β-actin. **(M, N)** ELISA quantification of ANGPT1, GABARAP, IL-18, and IL-1β in culture supernatants. After 48 h of siRNA transfection, cells were treated with recombinant human ANGPT1 (rhANGPT1; 200 ng/mL, 30 min), followed by LPS (1 μg/mL, 4 h) and nigericin (10 μM, 2 h), as indicated; unstimulated cells served as controls. Representative western blots are shown, and densitometric quantification was based on three independent transfection experiments. NLRP3, ANGPT1/GSDMD, and Caspase-1/GABARAP were detected on separate membranes with corresponding β-actin controls. Data are shown as mean ± SD (n = 3) and analyzed by one-way ANOVA followed by Tukey’s multiple comparisons test.

ANGPT1 knockdown efficiency was confirmed (**Supplementary Figure 2**). Under LPS plus nigericin stimulation, ANGPT1 knockdown reduced GABARAP expression, whereas recombinant human ANGPT1 (rhANGPT1) increased GABARAP levels (**Figures 5A–L**). ANGPT1 knockdown further enhanced NLRP3 expression, increased Caspase-1 processing and GSDMD-N accumulation, whereas rhANGPT1 partially attenuated these pyroptosis-associated molecular changes (**Figures 5A–L**). ELISA showed that ANGPT1 knockdown increased IL-1β and IL-18 secretion, whereas rhANGPT1 reduced their levels (**Figures 5M, N**). Together, these findings suggest that ANGPT1 may attenuate NLRP3 inflammasome-associated pyroptotic signaling under LPS plus nigericin stimulation, accompanied by restored GABARAP expression.

## 4 Discussion

In this study, we integrated pQTL-based MR, two-step MR, mediation analysis, DSS-induced colitis, and cell-based validation to investigate a putative ANGPT1–GABARAP axis in NLRP3 inflammasome-associated pyroptotic signaling. The MR screen prioritized GABARAP as a suggestive protective candidate for CD, and two-step MR supported a putative genetic relationship between ANGPT1 and GABARAP. In DSS-induced colitis and LPS plus nigericin-stimulated cell models, reduced ANGPT1 and GABARAP expression was accompanied by increased NLRP3 expression, Caspase-1 processing, GSDMD-N accumulation, and IL-1β/IL-18 release. Together, these findings suggest that ANGPT1–GABARAP signaling may be linked to inflammasome-associated pyroptotic signaling in intestinal inflammation, although direct molecular causality remains to be established.

The main novelty of this study lies in the integration of genetic prioritization with experimental validation to explore upstream signals associated with pyroptosis-related inflammatory pathways in CD. Previous studies have implicated the NLRP3–Caspase-1–GSDMD pathway in intestinal inflammation, innate immune activation, and epithelial barrier injury (4–8, 19). However, the upstream molecular signals that connect genetic susceptibility with aberrant inflammasome activation remain incompletely defined. By combining pQTL-based MR with DSS-induced colitis and cellular perturbation models, our study provides a genetically informed framework for prioritizing the ANGPT1–GABARAP axis as a candidate pathway associated with pyroptosis-related inflammatory signaling. From a translational perspective, this framework may help prioritize upstream molecular candidates for future biomarker development and target validation in CD.

The MR findings should be interpreted with caution. Although GABARAP showed an inverse association with CD risk and was retained as a suggestive MR candidate for downstream validation, this signal should be interpreted in the context of the 24-protein screen. The use of large-scale pQTL and GWAS resources enabled systematic candidate prioritization; however, the exposure and outcome datasets were derived from different populations, including deCODE, UKB-PPP, and UK Biobank-based CD GWAS data. Differences in ancestry background, allele frequency, linkage disequilibrium structure, and protein-regulatory architecture may introduce residual population heterogeneity or pleiotropy. Although sensitivity analyses, including MR-Egger intercept, heterogeneity testing, funnel plots, and leave-one-out analysis, did not indicate marked instability, these analyses cannot fully exclude all violations of MR assumptions. Therefore, the MR results should be considered hypothesis-generating and supportive of genetic prioritization rather than definitive proof of causality.

The two-step MR and mediation analysis further suggested a putative ANGPT1–GABARAP relationship associated with CD risk. Genetically predicted ANGPT1 was positively associated with GABARAP abundance, and mediation analysis suggested that GABARAP may partially mediate the genetically predicted ANGPT1–CD association. However, the estimated mediation proportion was limited and should not be interpreted as evidence of full mediation or a definitive causal hierarchy. Instead, these data support a partial statistical indirect association that requires further experimental validation. In particular, whether ANGPT1 directly regulates GABARAP expression, and whether GABARAP is required for the effect of ANGPT1 on NLRP3 inflammasome activation, remain unresolved.

*In vivo*, DSS-induced colitis was accompanied by reduced ANGPT1 and GABARAP expression and enhanced NLRP3 inflammasome-associated pyroptotic signaling. These changes occurred together with body weight loss, increased DAI scores, colon shortening, histological injury, elevated IL-1β/IL-18 levels, and increased LDH activity. These findings are consistent with the notion that reduced ANGPT1–GABARAP signaling may parallel inflammasome-related inflammatory injury. Nevertheless, DSS-induced colitis is primarily a model of epithelial injury and acute mucosal inflammation and does not fully reproduce the transmural and granulomatous inflammation characteristic of CD (20, 21). Thus, the DSS data should be interpreted as evidence from an experimental intestinal inflammation model rather than as direct evidence of CD-specific pathology.

The cell-based experiments further supported a link between ANGPT1–GABARAP signaling and NLRP3 inflammasome-associated pyroptotic signaling. In THP-1-derived macrophages and NCM460 epithelial cells, GABARAP knockdown enhanced LPS plus nigericin-induced increases in NLRP3, Caspase-1 (p10), GSDMD-N, IL-1β, and IL-18. ANGPT1 knockdown reduced GABARAP expression and enhanced pyroptosis-associated molecular changes, whereas rhANGPT1 partially attenuated these responses, accompanied by restored GABARAP expression. These findings support an association between ANGPT1 treatment, GABARAP restoration, and reduced inflammasome-associated pyroptotic signaling. However, they do not establish that ANGPT1 acts exclusively through GABARAP. Critical rescue experiments, such as testing whether rhANGPT1 still attenuates NLRP3 inflammasome-associated signaling when GABARAP is silenced, will be required to determine whether GABARAP is necessary for ANGPT1-mediated effects.

Mechanistically, GABARAP belongs to the ATG8 family and is involved in autophagy-related membrane trafficking and autophagosome–lysosome fusion (22, 23), processes that may influence inflammasome activation, organelle stress responses, and degradation of inflammatory signaling components. However, the present study did not define the direct molecular route linking GABARAP to NLRP3 regulation. Potential mechanisms include autophagy-mediated degradation of inflammasome components, lysosomal homeostasis, mitochondrial stress regulation, or modulation of upstream priming pathways such as NF-κB (19, 24). Similarly, the mechanism by which ANGPT1 influences GABARAP expression remains to be clarified. Future studies using co-immunoprecipitation, autophagy flux assays, NF-κB pathway inhibition or activation assays, and loss-of-function rescue designs will be important to define the direct molecular relationship among ANGPT1, GABARAP, and NLRP3 inflammasome activation.

Several limitations should be acknowledged. First, the MR analyses rely on core assumptions and may still be influenced by residual pleiotropy, cross-population heterogeneity, and incomplete ancestry matching. In addition, the GABARAP signal should be interpreted as suggestive in the context of the 24-protein screen, and the mediation estimate reflects partial statistical mediation rather than a definitive biological causal chain. Second, the experimental models have inherent limitations. DSS-induced colitis primarily reflects epithelial injury and mucosal inflammation rather than CD-specific transmural pathology, and THP-1-derived macrophages, NCM460 cells, and LPS plus nigericin stimulation do not fully reproduce primary intestinal immune or epithelial cells or the complex CD inflammatory microenvironment. Third, the present data do not establish the direct molecular mechanism linking ANGPT1, GABARAP, and NLRP3 inflammasome activation, nor do they prove that ANGPT1 acts exclusively through GABARAP. Direct interaction assays, autophagy flux analyses, NF-κB pathway inhibition or activation assays, and rescue experiments will be required. Finally, further validation in primary cells, intestinal organoids, patient-derived tissues, and direct barrier-function assays is needed to define the translational relevance of this axis.

In summary, this study supports a genetically informed association between the ANGPT1–GABARAP axis and NLRP3 inflammasome-associated pyroptotic signaling in intestinal inflammation. Rather than establishing a definitive causal mechanism, the findings nominate ANGPT1–GABARAP signaling as a candidate pathway that may connect genetic susceptibility with inflammasome-related inflammatory injury. Further mechanistic and translational studies are warranted to determine whether this axis has therapeutic relevance in CD.

## Data Availability

The pQTL datasets analyzed in this study were obtained from the deCODE cohort and the UKB-PPP. Crohn’s disease GWAS summary statistics were obtained from the GWAS Catalog. Derived MR results, mediation analysis results, and experimental data supporting the findings of this study are available within the article and its Supplementary Material. Additional source data and analysis code are available from the corresponding author upon reasonable request.

## Supplementary material

Supplementary Figure 1 Knockdown efficiency of GABARAP-targeting siRNAs in THP-1-derived macrophages and NCM460 cells.

Supplementary Figure 2 Knockdown efficiency of ANGPT1-targeting siRNAs in THP-1-derived macrophages and NCM460 cells.
