# Supplementary figures and images for: ANGPT1–GABARAP axis modulates NLRP3 inflammasome–mediated pyroptosis in Crohn’s disease
Source: Front Immunol. 2026 Jun 10;17:1867825. doi: 10.3389/fimmu.2026.1867825 (PMC13290549; doi:10.3389/fimmu.2026.1867825)

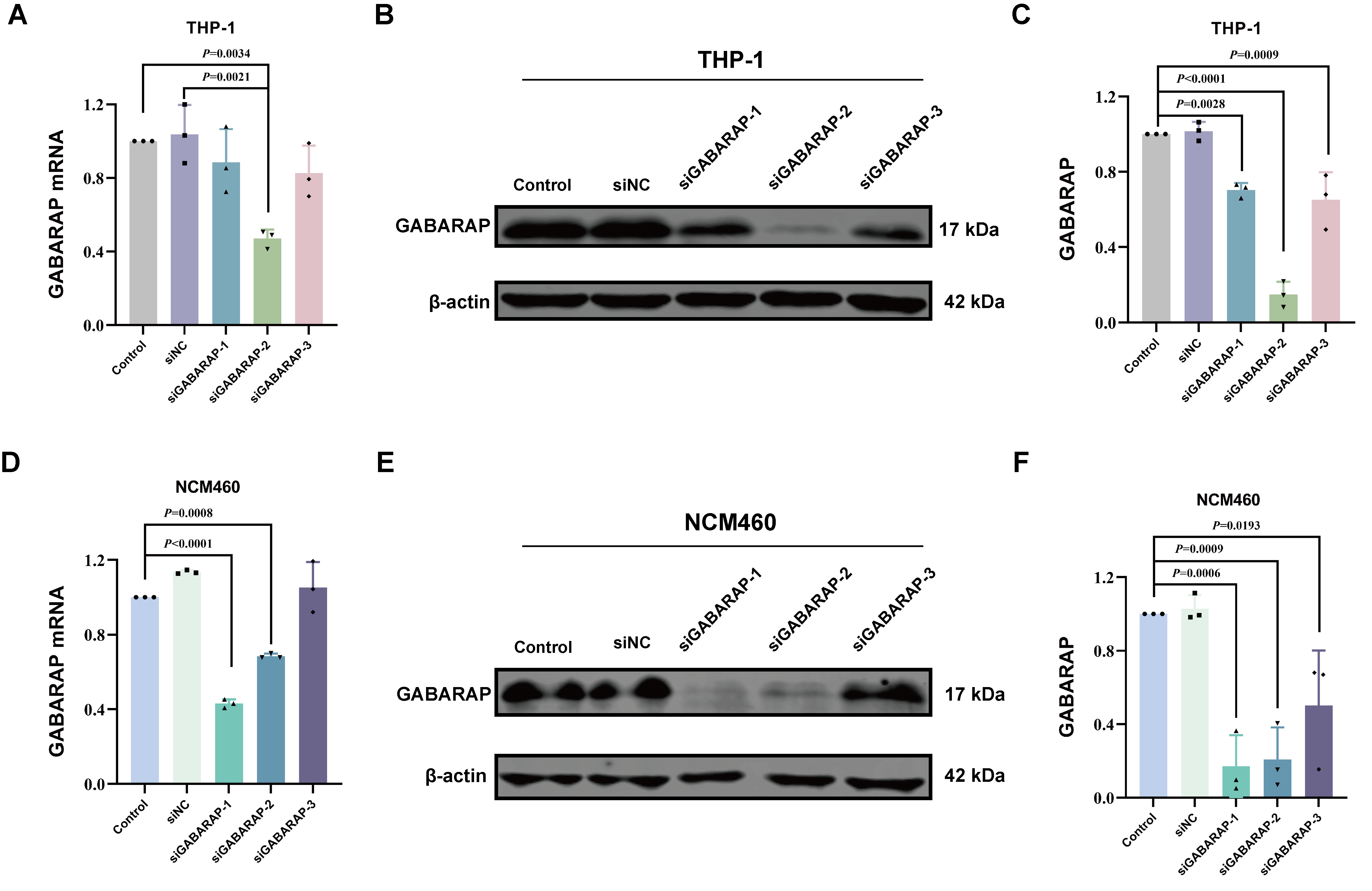

Supplement: Supplementary Figure 1 — Knockdown efficiency of GABARAP-targeting siRNAs in THP-1-derived macrophages and NCM460 cells. [file Image1.tif]

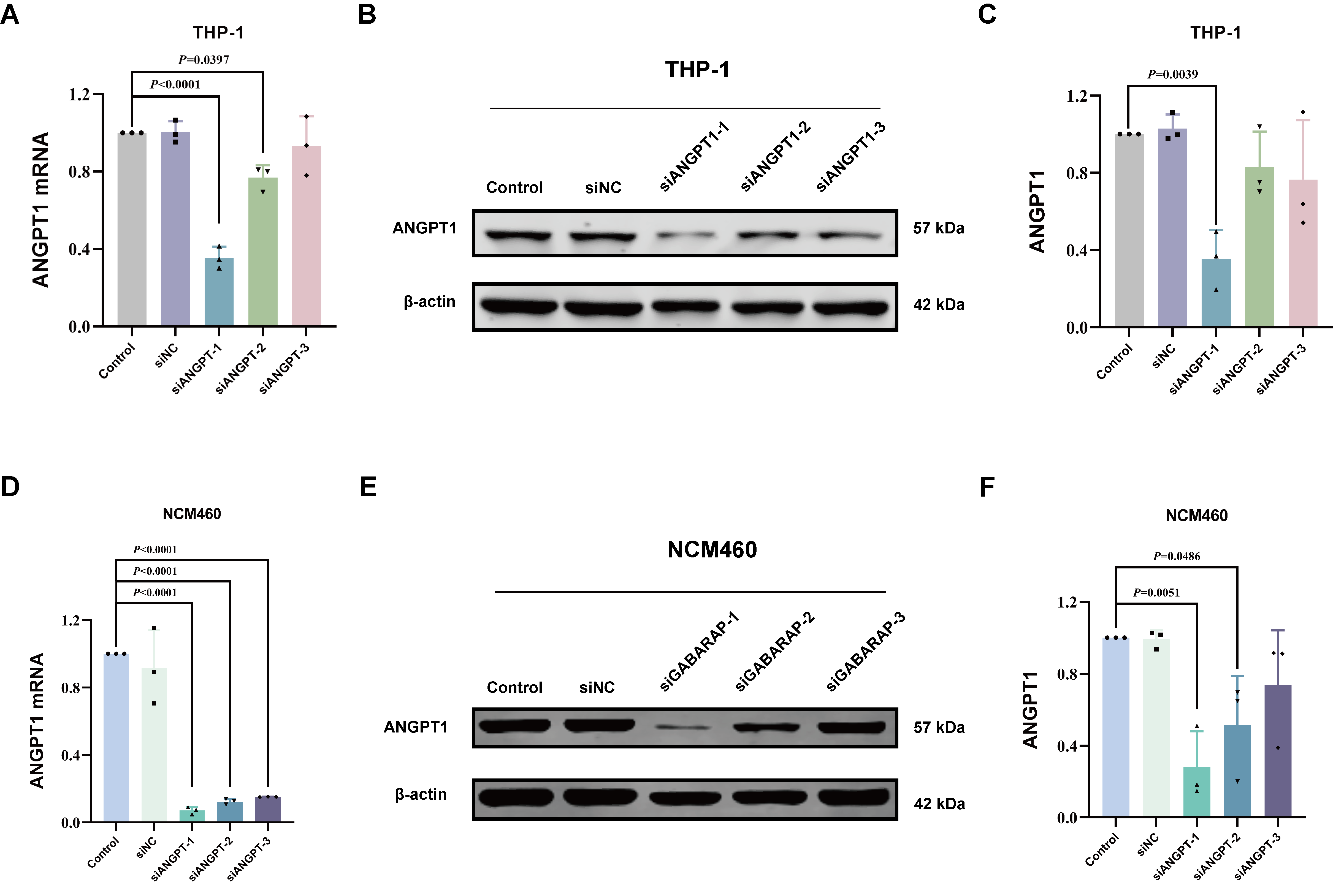

Supplement: Supplementary Figure 2 — Knockdown efficiency of ANGPT1-targeting siRNAs in THP-1-derived macrophages and NCM460 cells. [file Image2.tif]

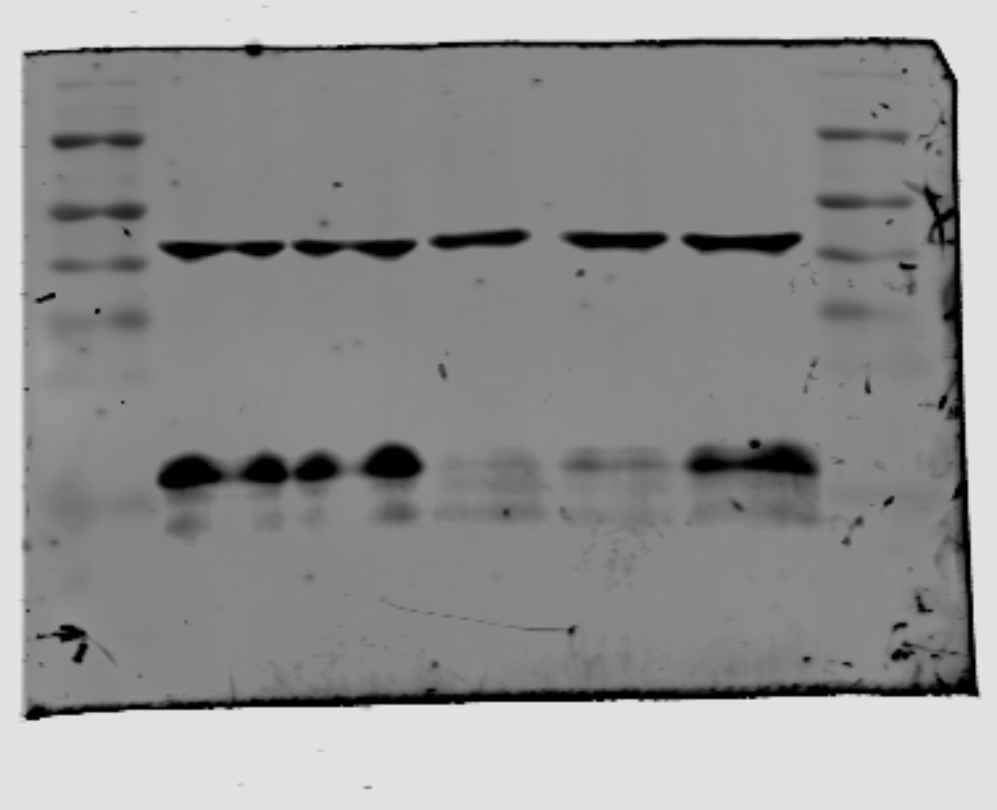

Supplement: Supplementary file 4 [file DataSheet2.zip › WB/Fig.S1/NCM460V GABARAPsiRNA/2025-10-19-NCM460-GABARAP siRNA.tif]

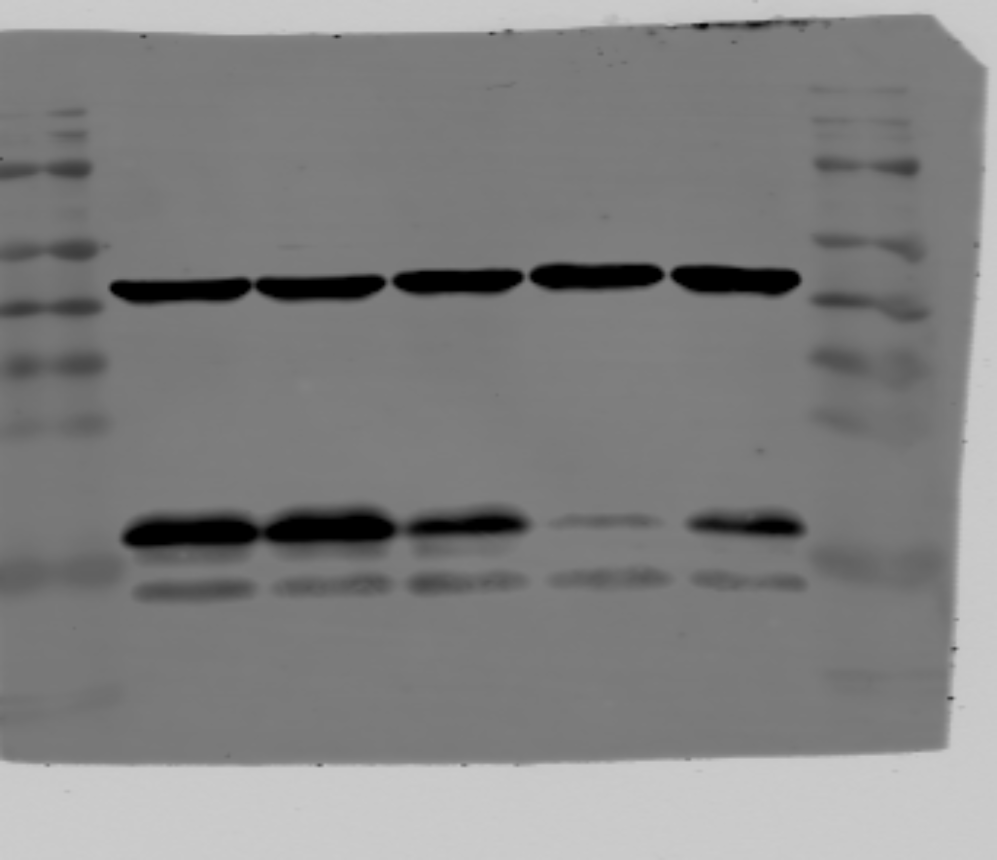

Supplement: Supplementary file 4 [file DataSheet2.zip › WB/Fig.S1/THP-1-GABARAP siRNA/2025-09-23-THP1-GABARAP siRNA.tif]

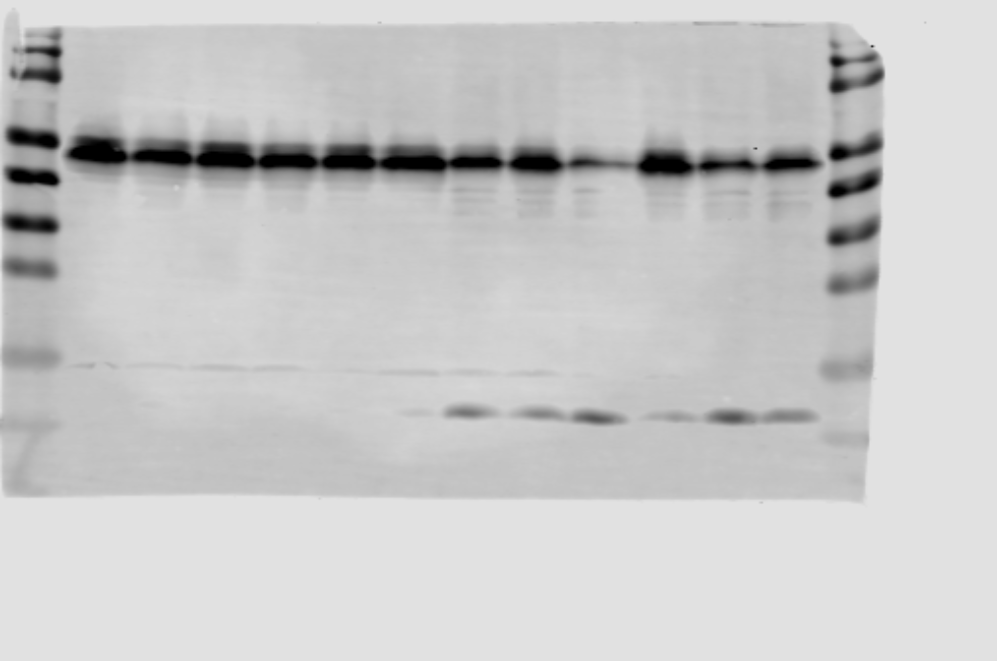

Supplement: Supplementary file 4 [file DataSheet2.zip › WB/Fig3/2025-11-18-DSS-Caspase1.tif]

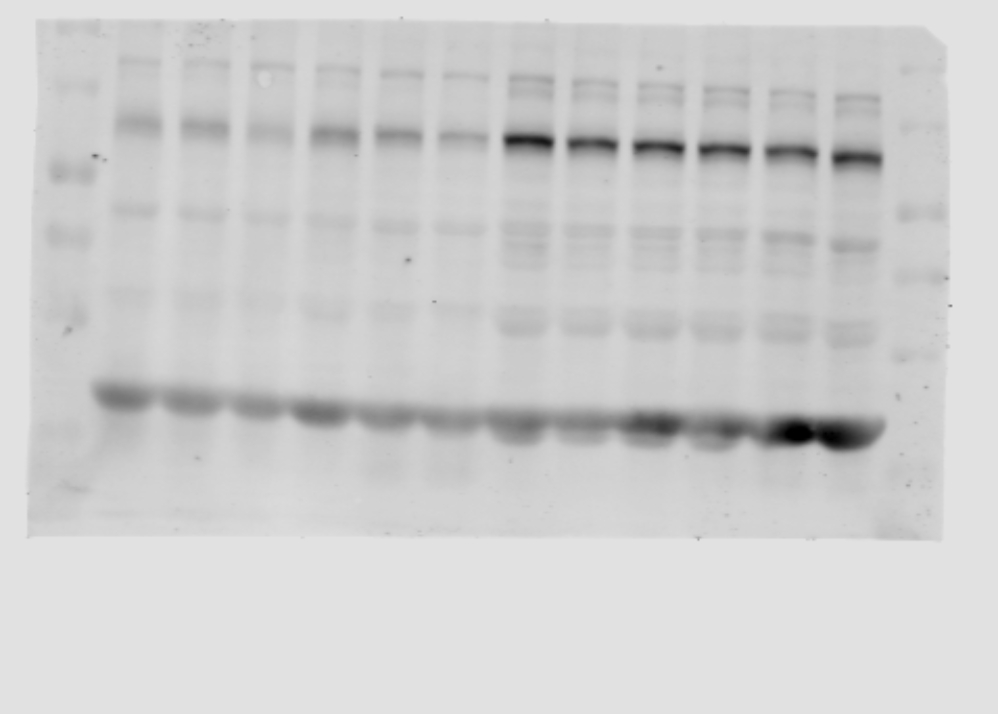

Supplement: Supplementary file 4 [file DataSheet2.zip › WB/Fig3/2025-11-24-DSS-NLRP3-Actin.jpg]

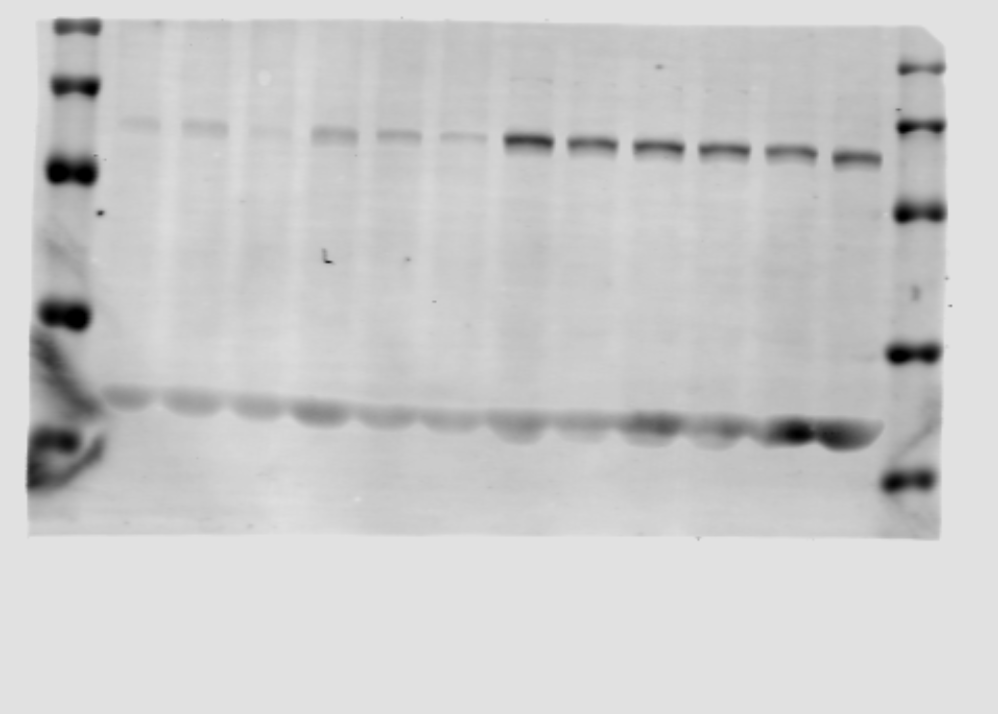

Supplement: Supplementary file 4 [file DataSheet2.zip › WB/Fig3/2025-11-24-DSS-NLRP3-Actin.tif]

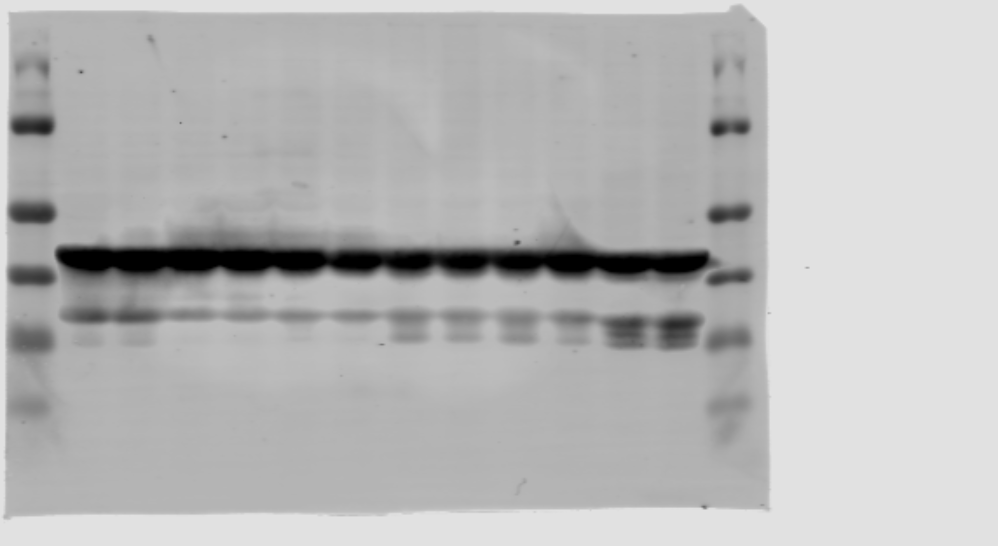

Supplement: Supplementary file 4 [file DataSheet2.zip › WB/Fig3/2025-12-19-DSS-Actin.tif]

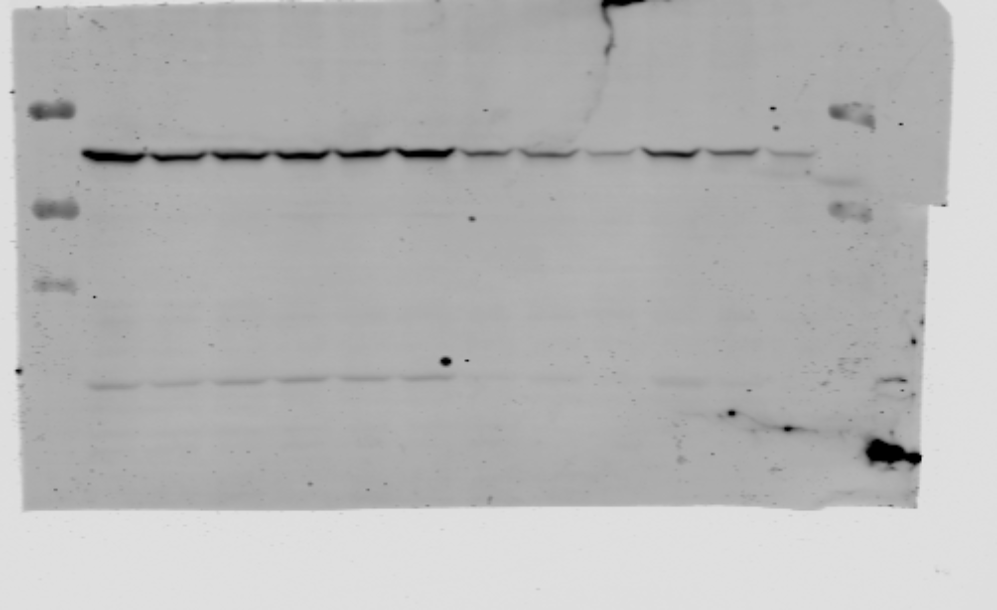

Supplement: Supplementary file 4 [file DataSheet2.zip › WB/Fig3/2025-12-19-DSS-ANGPT.tif]

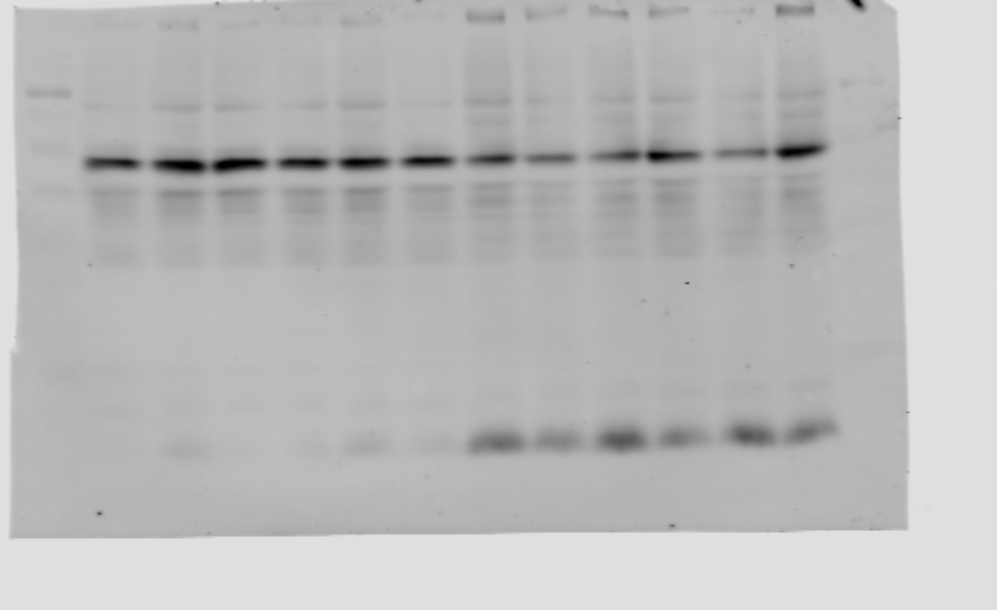

Supplement: Supplementary file 4 [file DataSheet2.zip › WB/Fig3/2025-12-19-DSS-caspase1.tif]

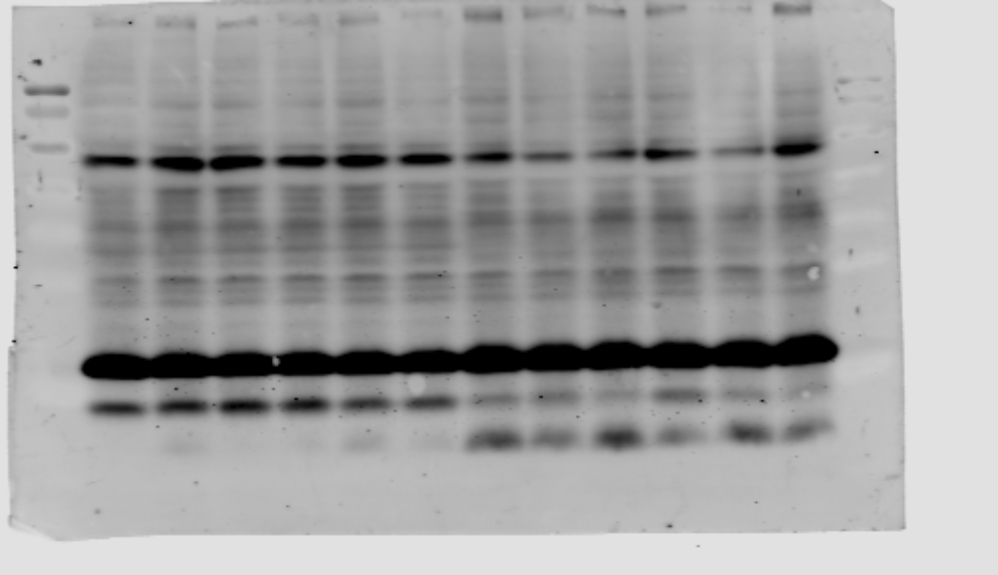

Supplement: Supplementary file 4 [file DataSheet2.zip › WB/Fig3/2025-12-19-DSS-GABARAP.tif]

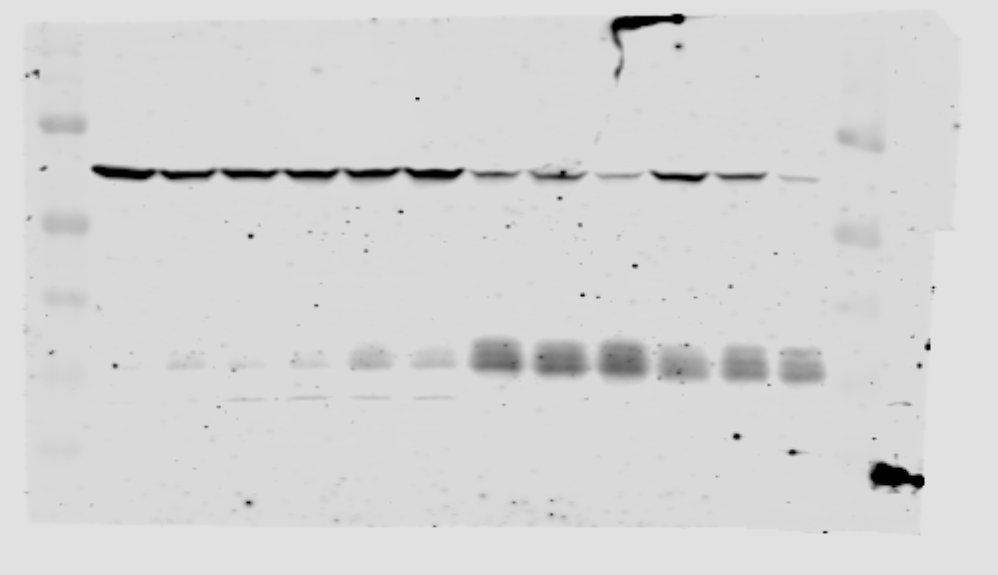

Supplement: Supplementary file 4 [file DataSheet2.zip › WB/Fig3/2025-12-19-DSS-GSDMD.tif]

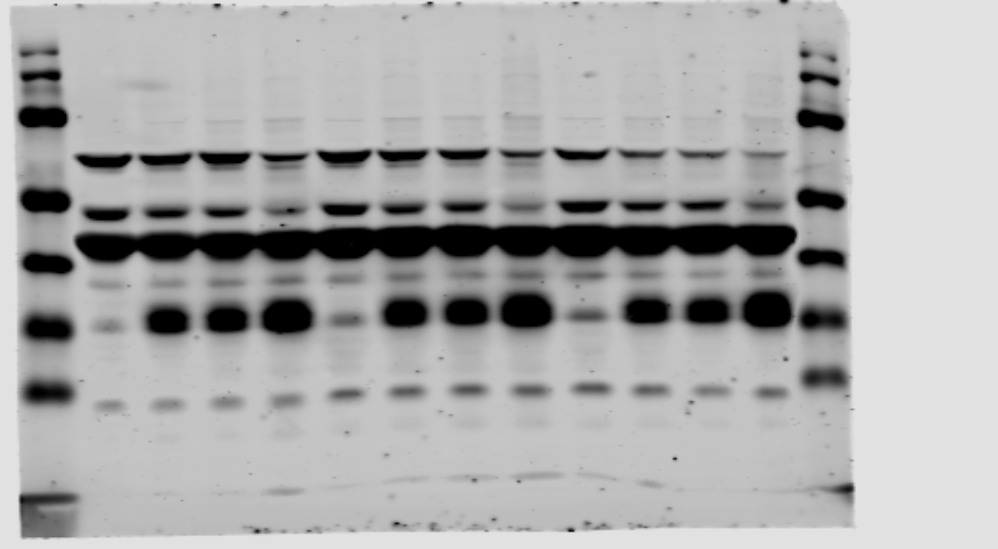

Supplement: Supplementary file 4 [file DataSheet2.zip › WB/Fig4/NCM460-LPS+N-siGABARAP-1/NCM460-LPS-Nigerin-siGABARAP-Actin.tif]

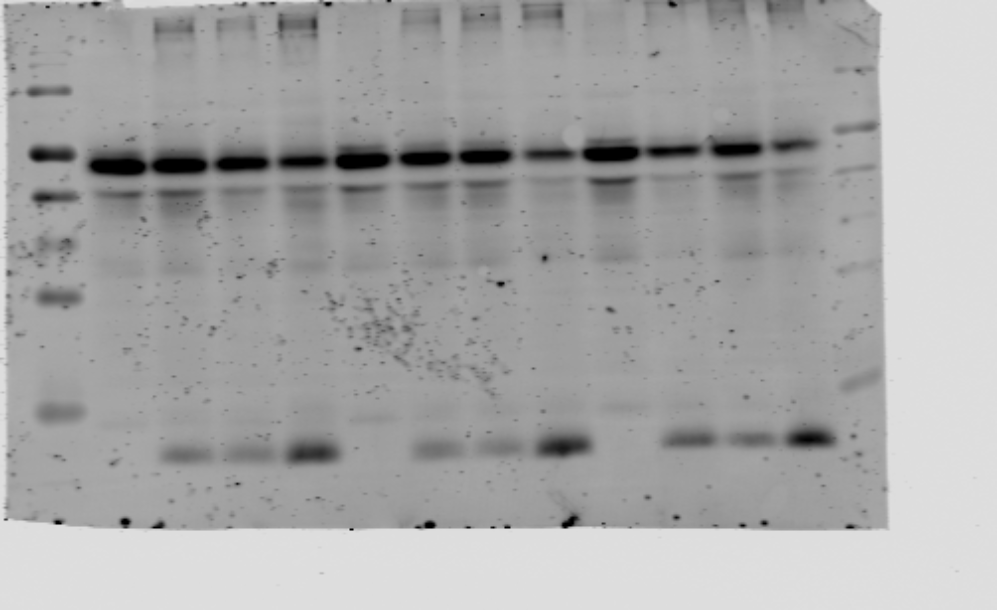

Supplement: Supplementary file 4 [file DataSheet2.zip › WB/Fig4/NCM460-LPS+N-siGABARAP-1/NCM460-LPS-Nigerin-siGABARAP-Caspase1.tif]

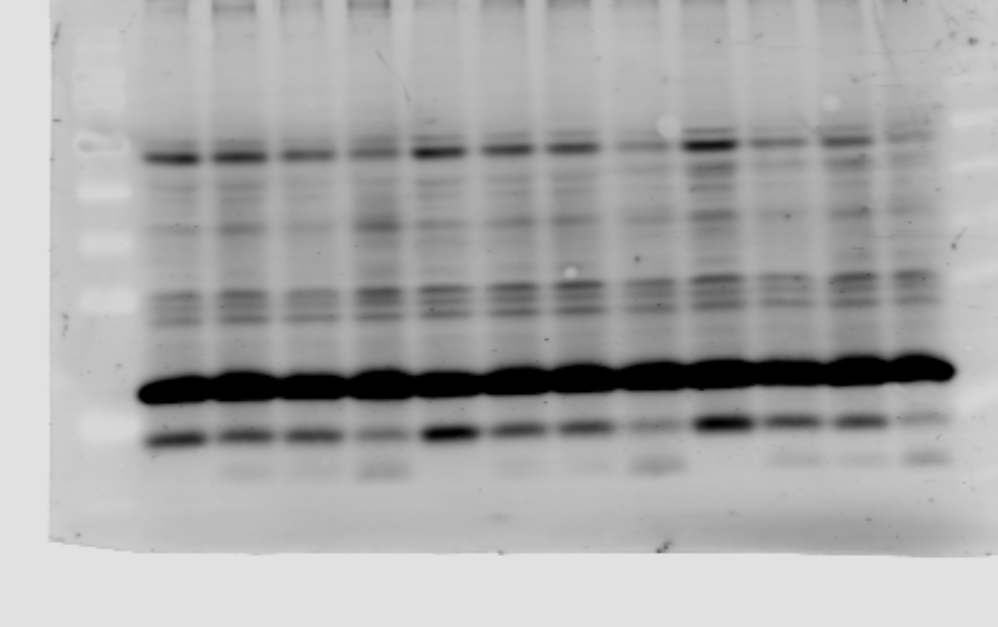

Supplement: Supplementary file 4 [file DataSheet2.zip › WB/Fig4/NCM460-LPS+N-siGABARAP-1/NCM460-LPS-Nigerin-siGABARAP-GABARAP.tif]

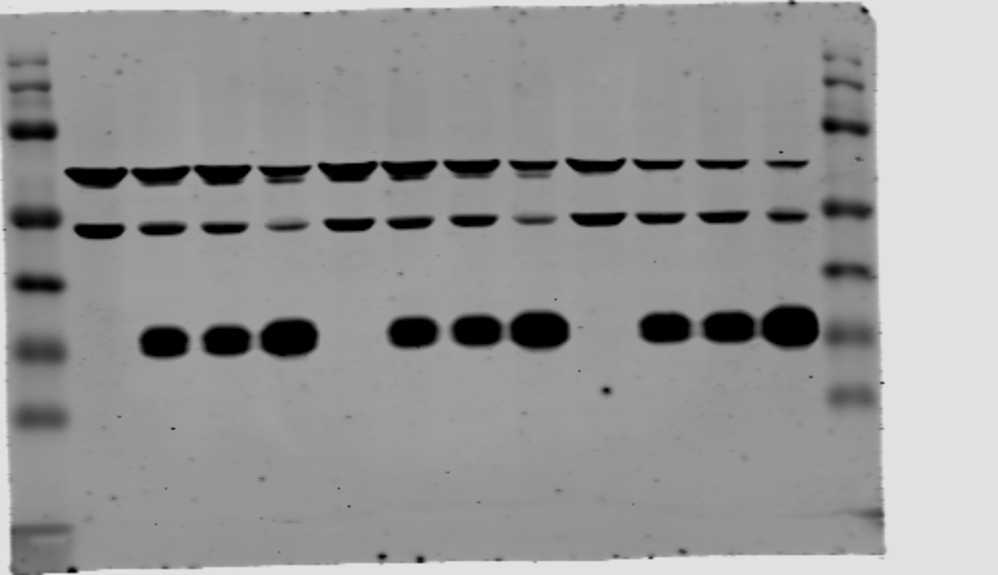

Supplement: Supplementary file 4 [file DataSheet2.zip › WB/Fig4/NCM460-LPS+N-siGABARAP-1/NCM460-LPS-Nigerin-siGABARAP-GSDMD.tif]

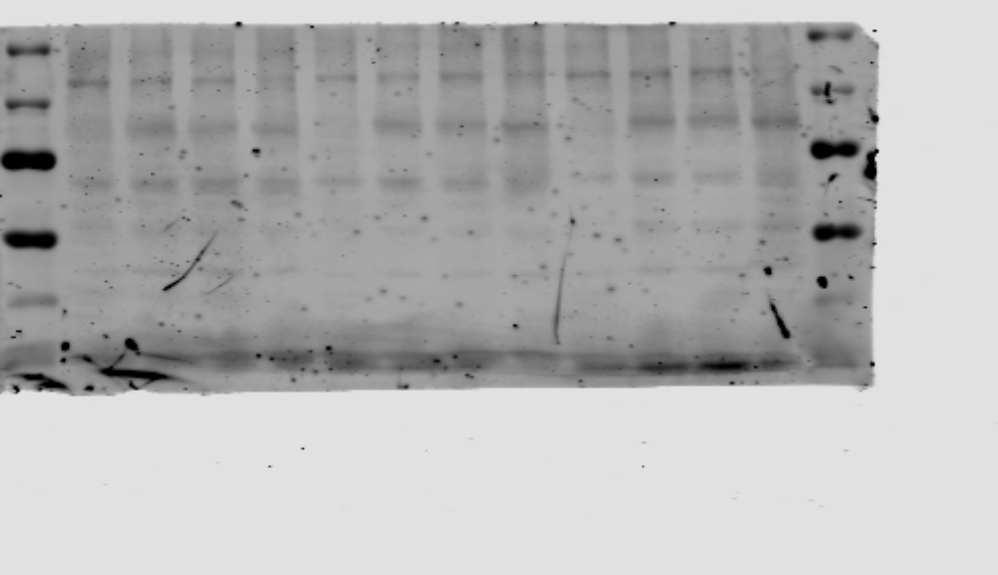

Supplement: Supplementary file 4 [file DataSheet2.zip › WB/Fig4/NCM460-LPS+N-siGABARAP-1/NCM460-LPS-Nigerin-siGABARAP-NLRP3.tif]

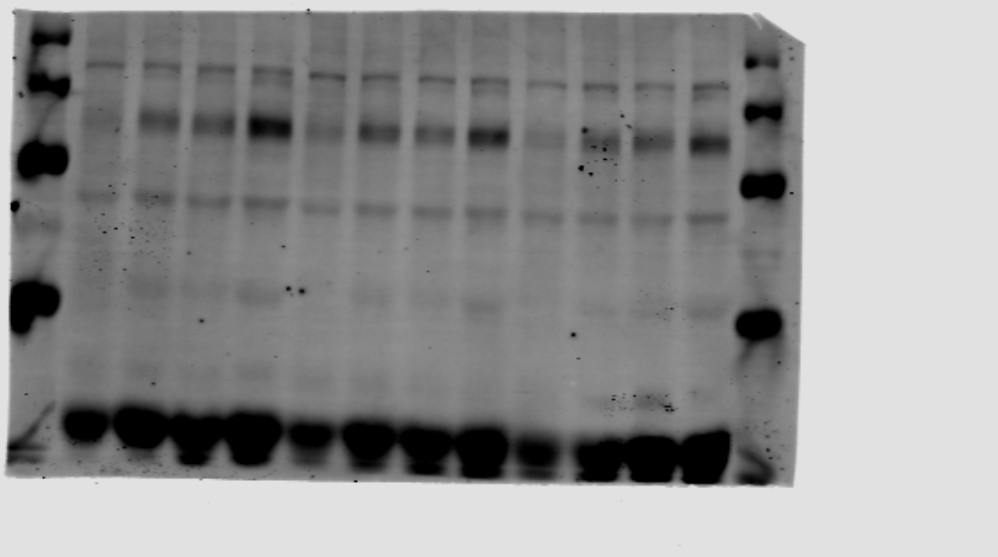

Supplement: Supplementary file 4 [file DataSheet2.zip › WB/Fig4/THP1-LPS+N-siGABARAP-1/2025-11-28-LPS-Nigerin-siGABARAP-NLRP3.tif]

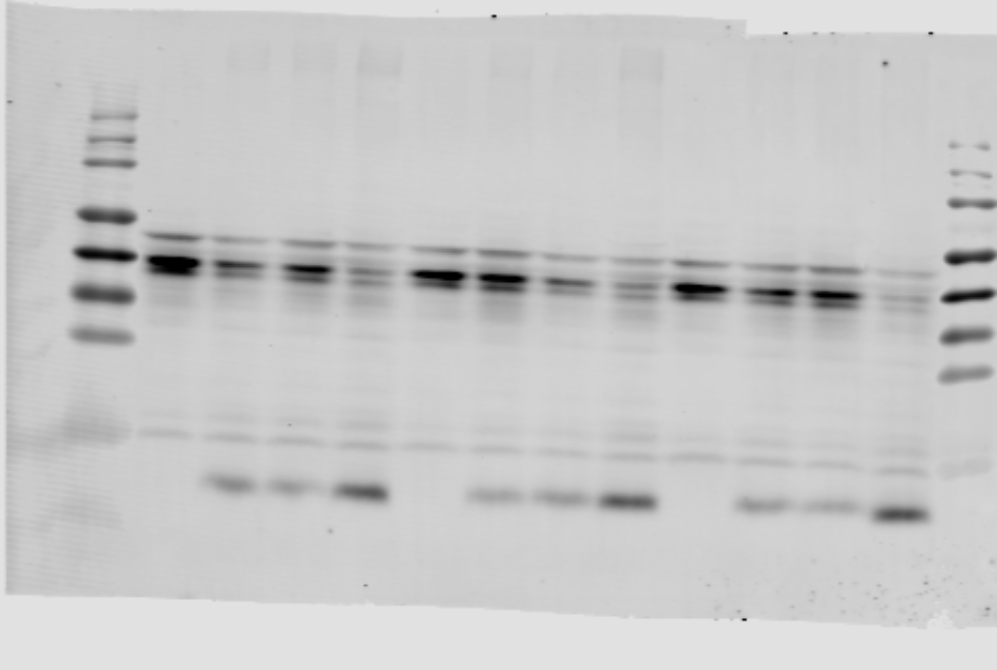

Supplement: Supplementary file 4 [file DataSheet2.zip › WB/Fig4/THP1-LPS+N-siGABARAP-1/2025-11-30-LPS-Nigerin-siGABARAP-Caspase1.tif]

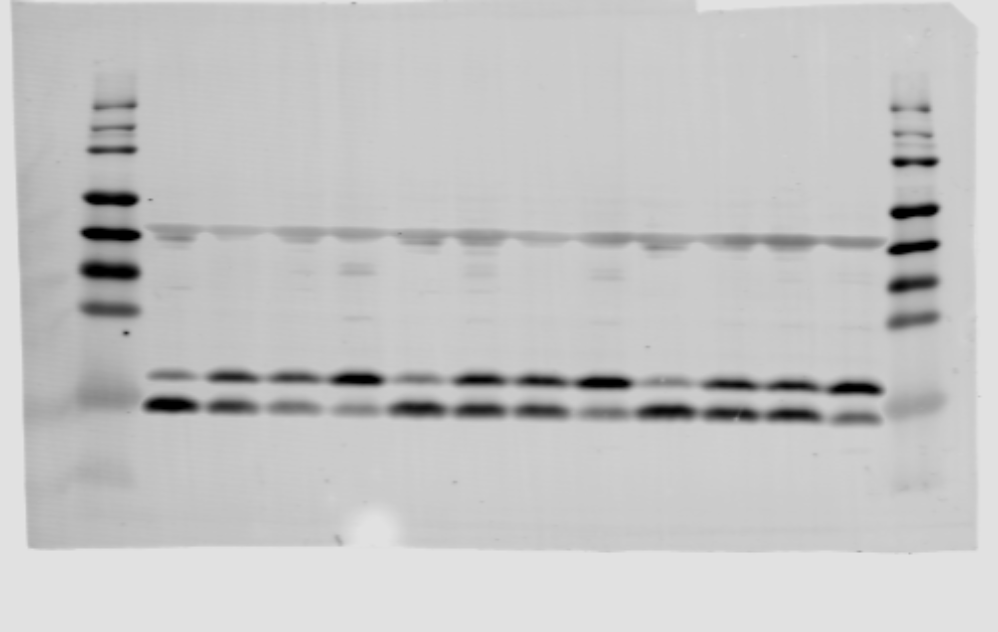

Supplement: Supplementary file 4 [file DataSheet2.zip › WB/Fig4/THP1-LPS+N-siGABARAP-1/2025-11-30-LPS-Nigerin-siGABARAP-GABARAP.tif]

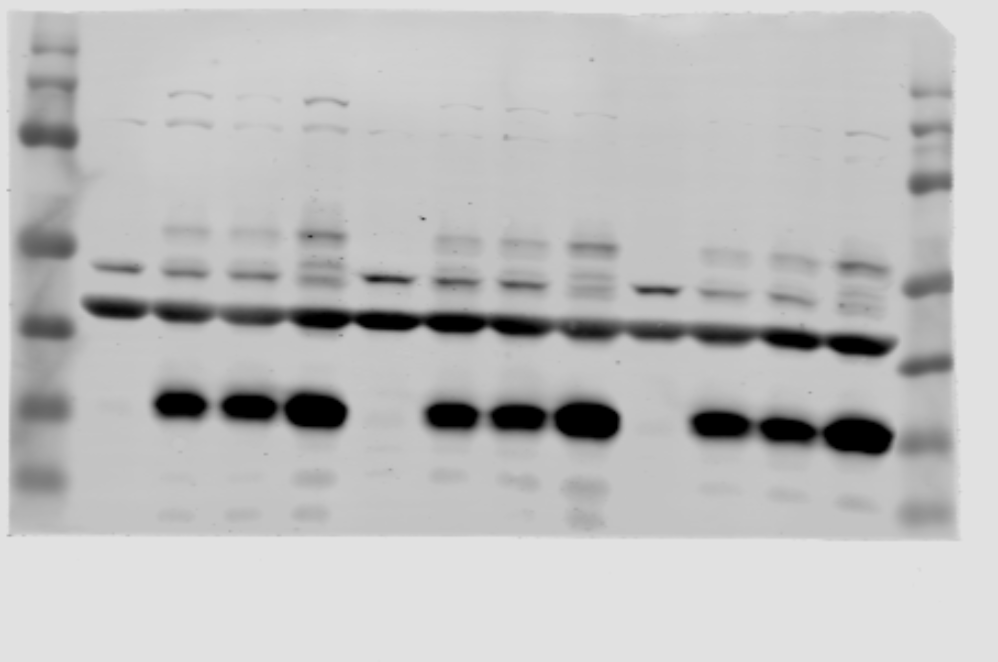

Supplement: Supplementary file 4 [file DataSheet2.zip › WB/Fig4/THP1-LPS+N-siGABARAP-1/2025-11-30-LPS-Nigerin-siGABARAP-GSDMD.tif]

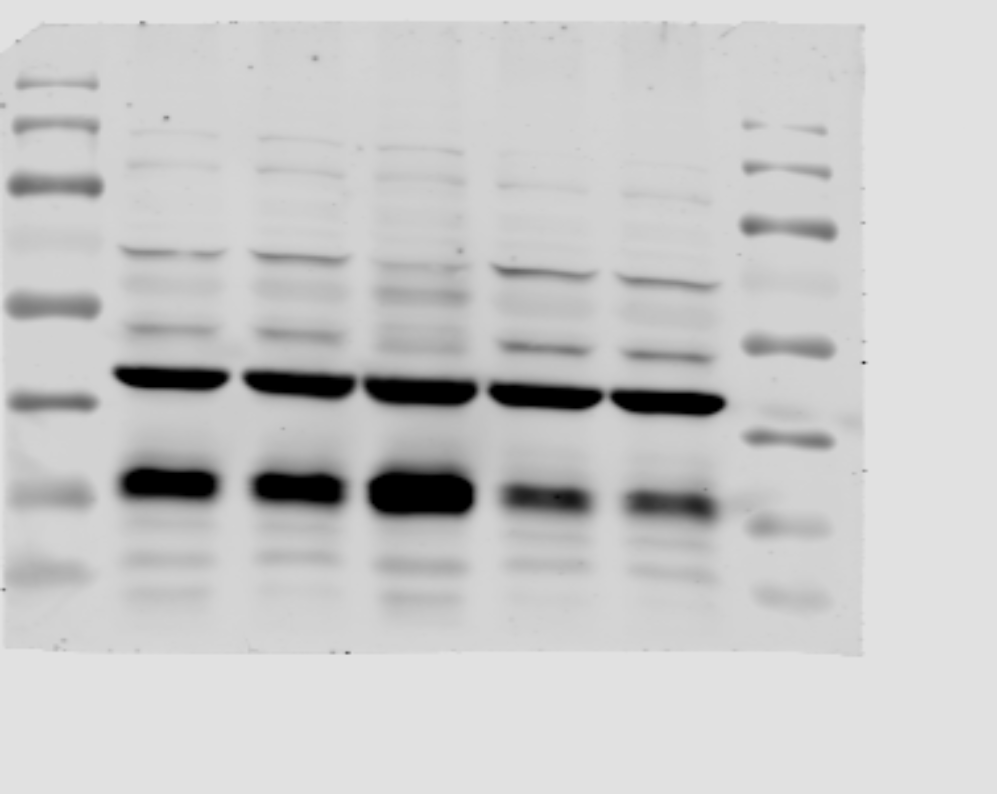

Supplement: Supplementary file 4 [file DataSheet2.zip › WB/Fig5/NCM460-LPS+Niger+siANGPT-1/NCM460-siANGPT1-Actin.tif.tif]

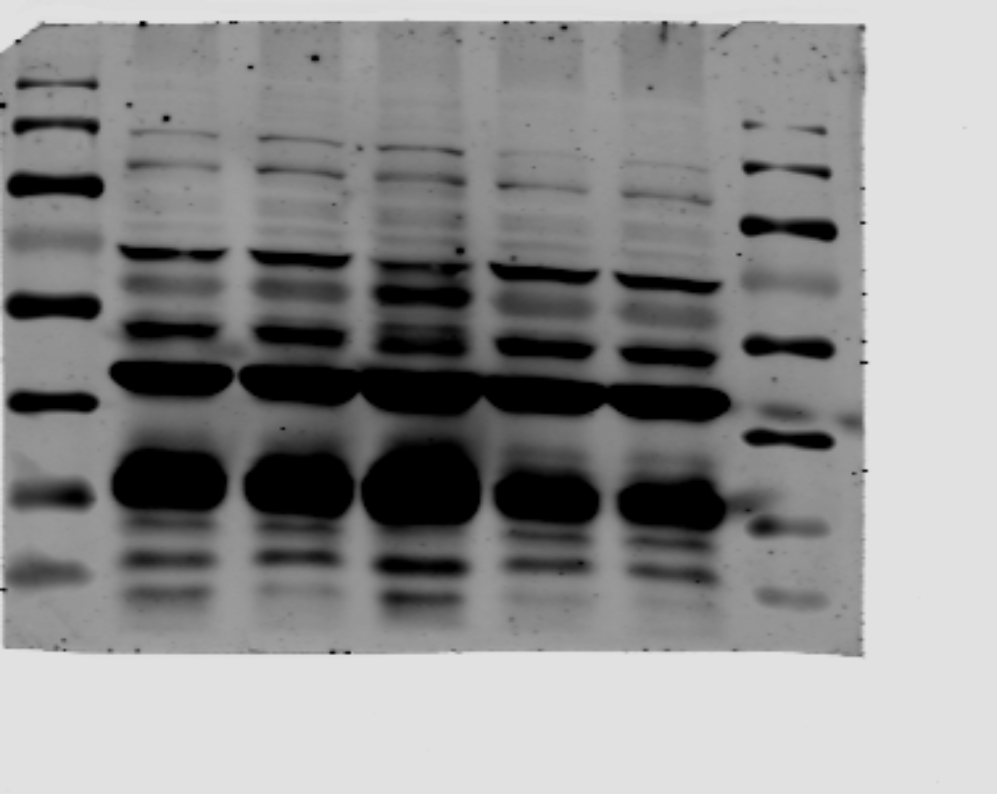

Supplement: Supplementary file 4 [file DataSheet2.zip › WB/Fig5/NCM460-LPS+Niger+siANGPT-1/NCM460-siANGPT1-ANGPT1.tif]

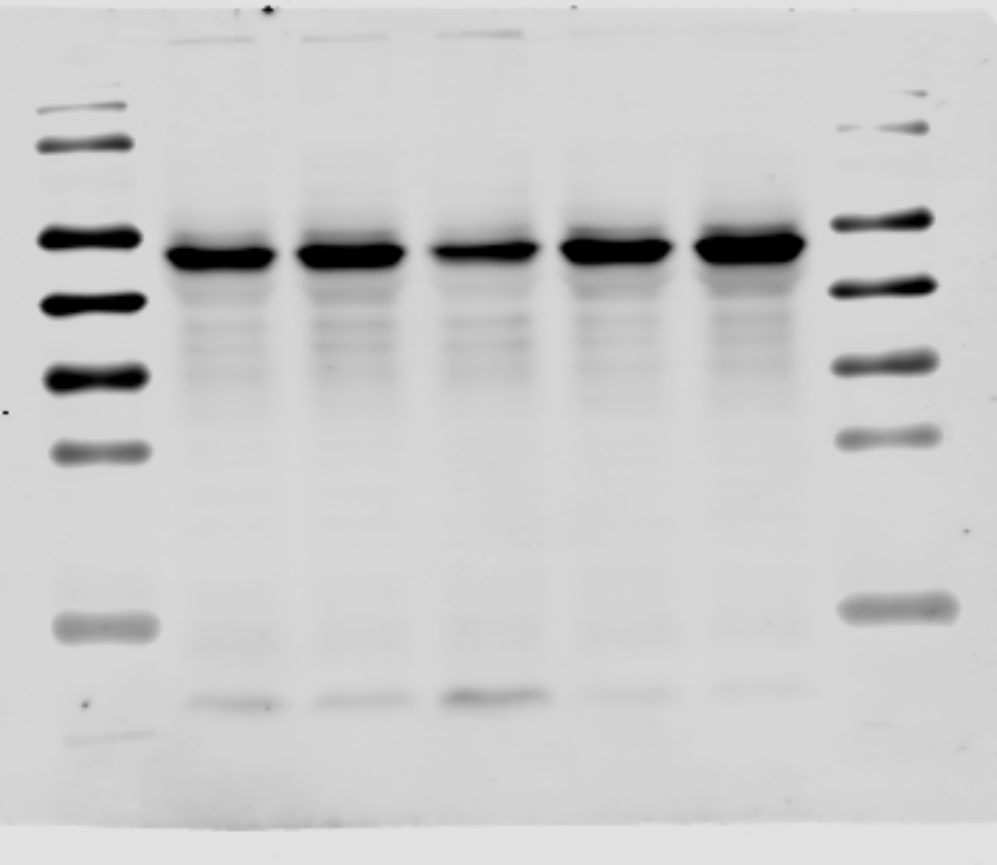

Supplement: Supplementary file 4 [file DataSheet2.zip › WB/Fig5/NCM460-LPS+Niger+siANGPT-1/NCM460-siANGPT1-Caspase.tif]

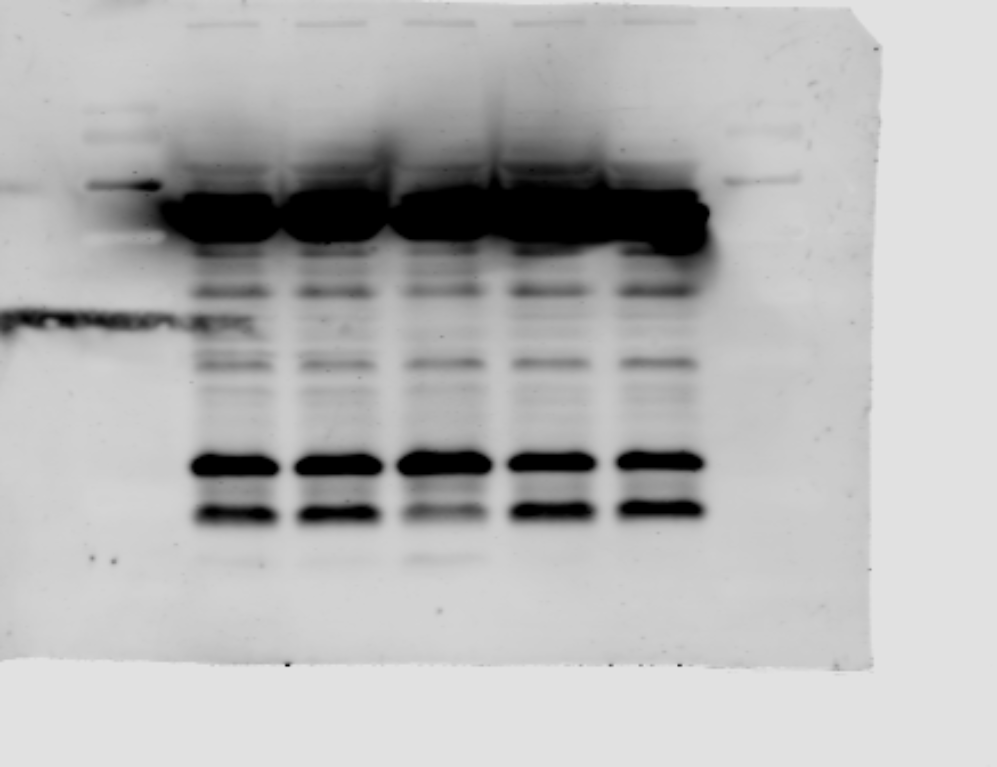

Supplement: Supplementary file 4 [file DataSheet2.zip › WB/Fig5/NCM460-LPS+Niger+siANGPT-1/NCM460-siANGPT1-GABARAP.tif]

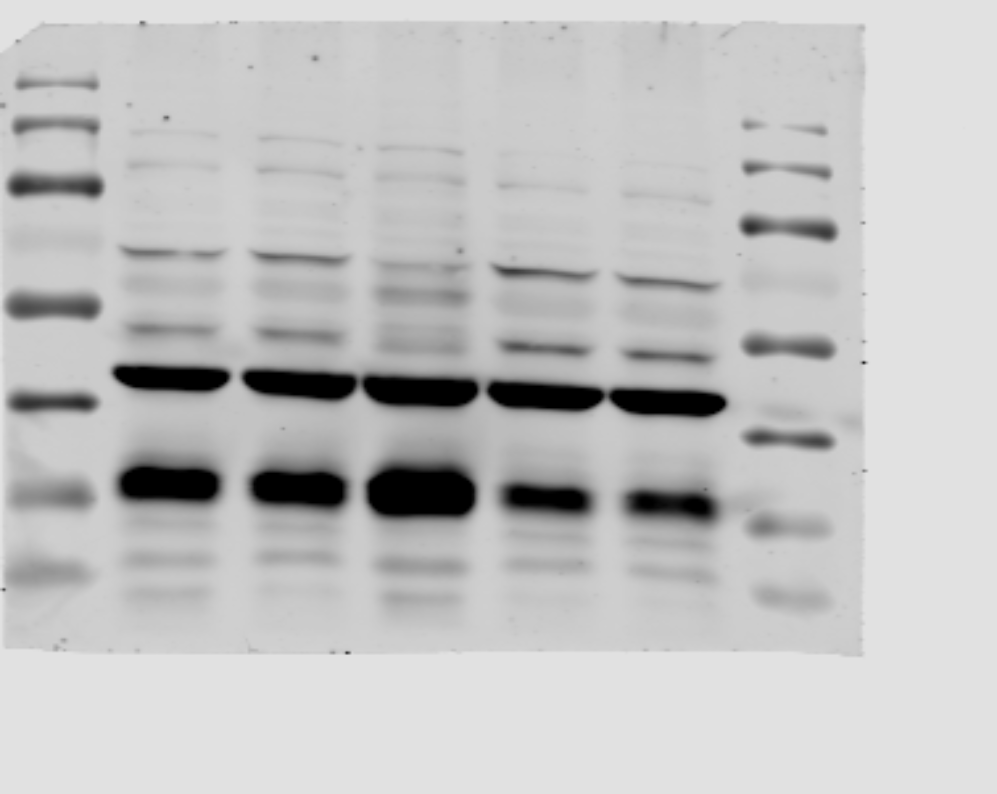

Supplement: Supplementary file 4 [file DataSheet2.zip › WB/Fig5/NCM460-LPS+Niger+siANGPT-1/NCM460-siANGPT1-GSDMD.tif.tif]

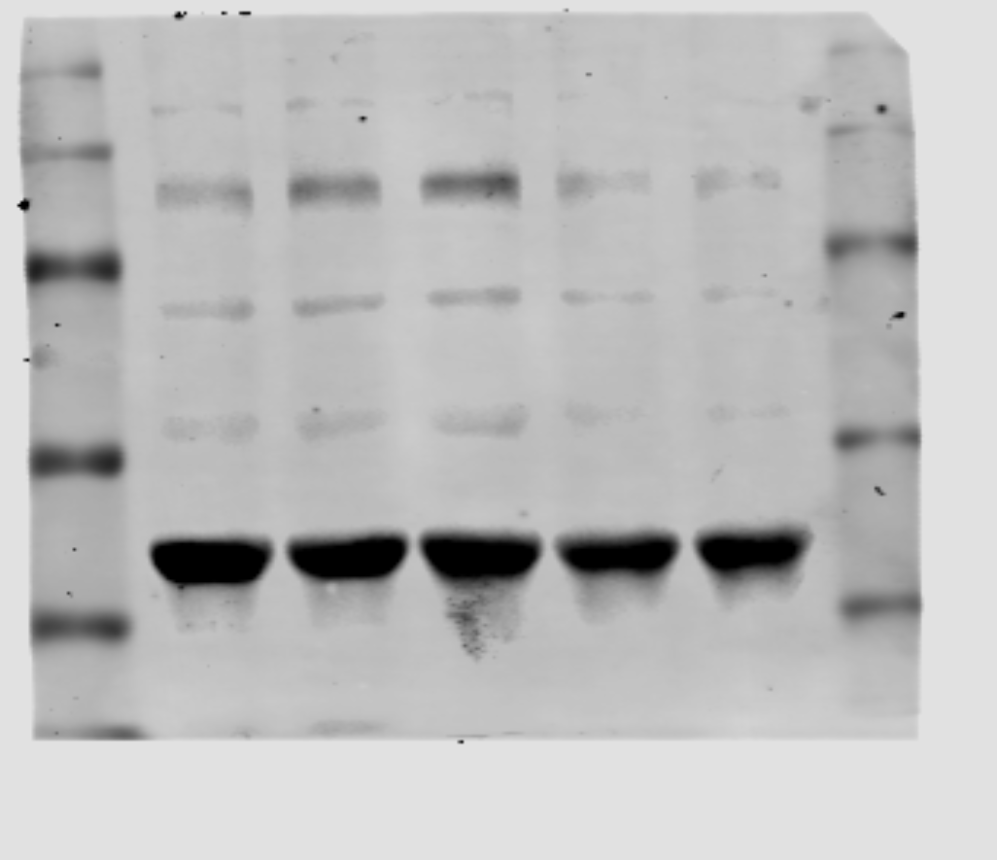

Supplement: Supplementary file 4 [file DataSheet2.zip › WB/Fig5/NCM460-LPS+Niger+siANGPT-1/NCM460-siANGPT1-NLRP3.tif]

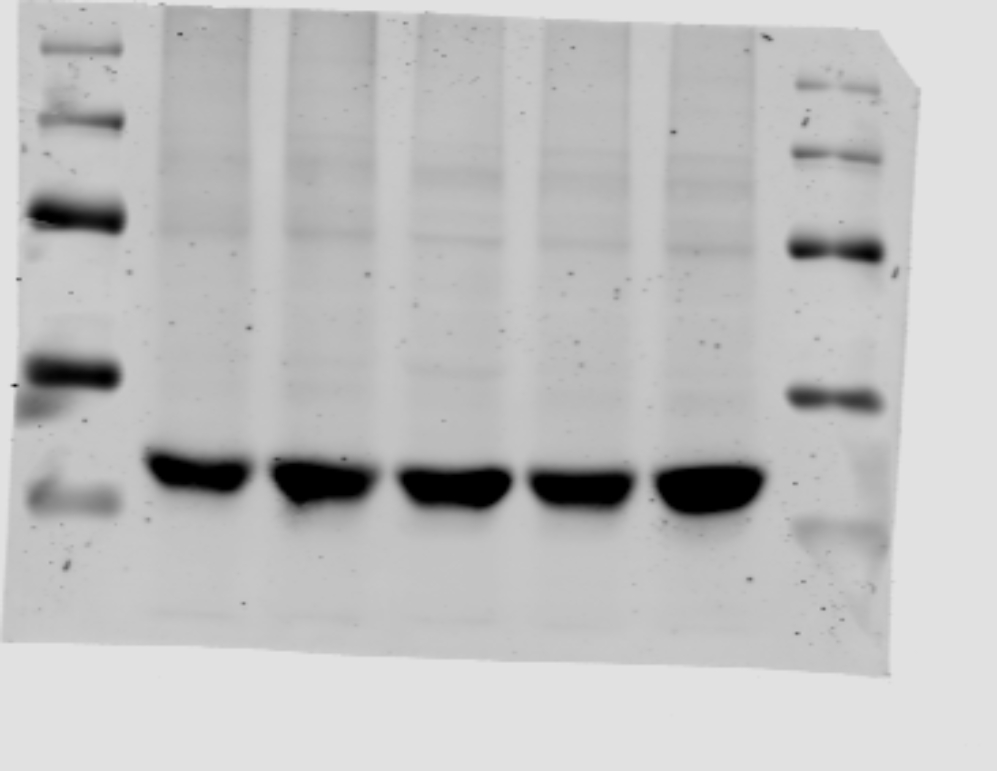

Supplement: Supplementary file 4 [file DataSheet2.zip › WB/Fig5/THP1-LPS+Niger+siANGPT/2025-12-03-LPS-N-siANGPT-NLRP3 actin-2.jpg]

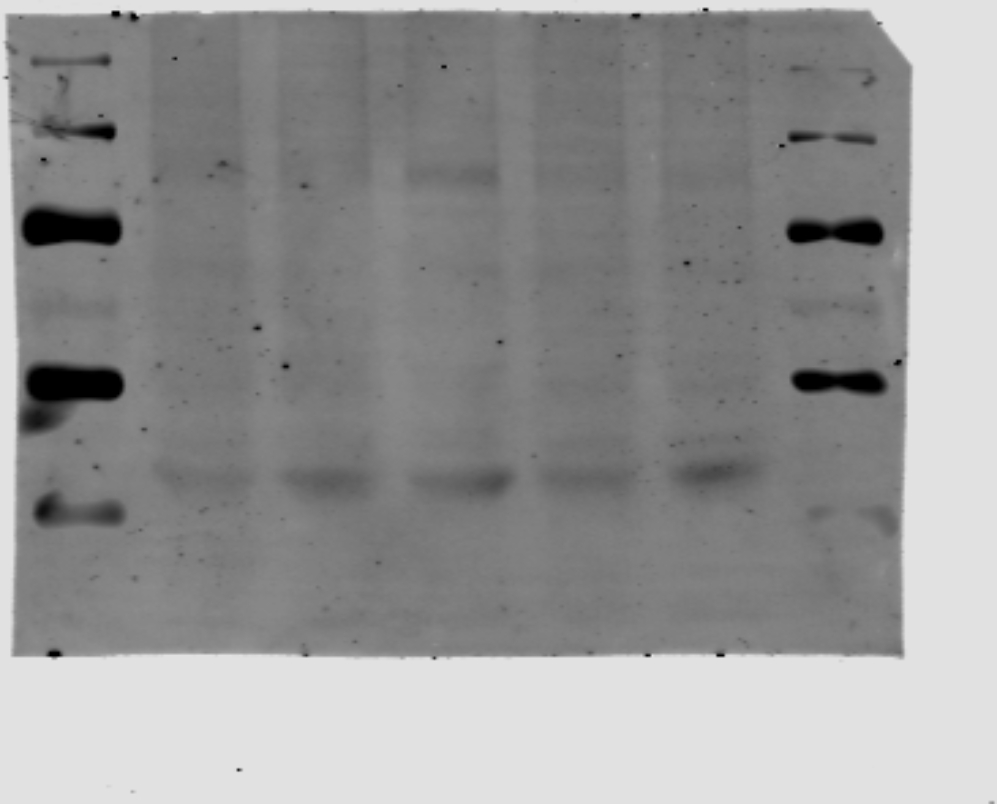

Supplement: Supplementary file 4 [file DataSheet2.zip › WB/Fig5/THP1-LPS+Niger+siANGPT/2025-12-03-LPS-N-siANGPT-NLRP3-2.jpg]

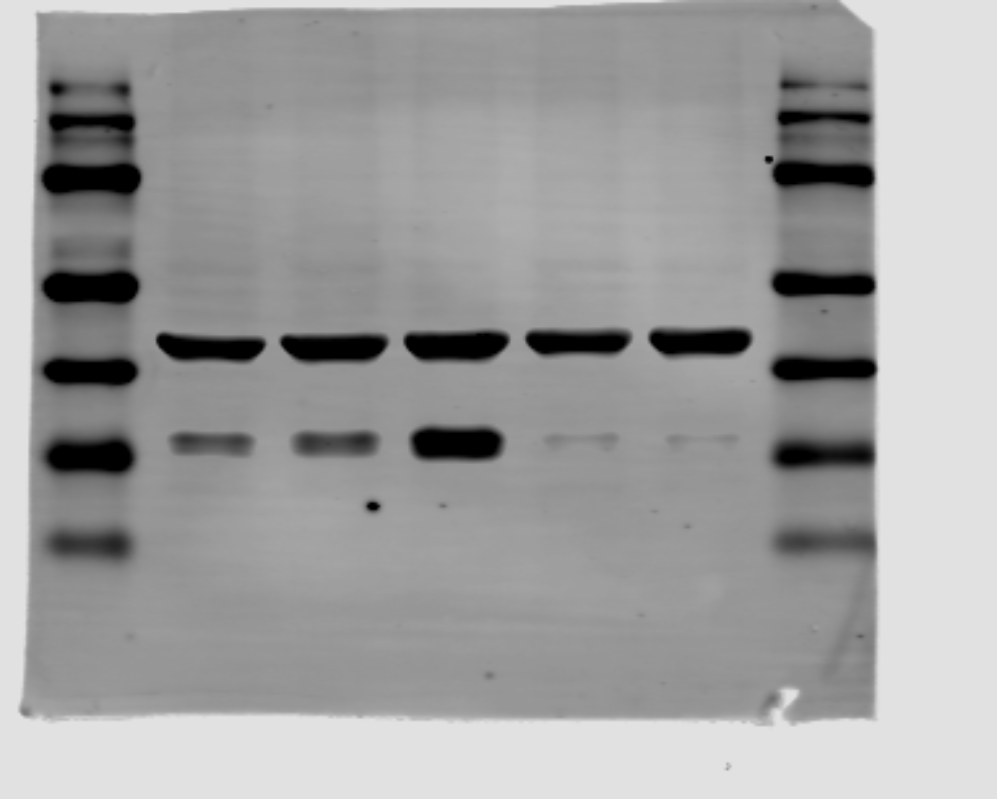

Supplement: Supplementary file 4 [file DataSheet2.zip › WB/Fig5/THP1-LPS+Niger+siANGPT/THP1-siANGPT1-actin.tif]

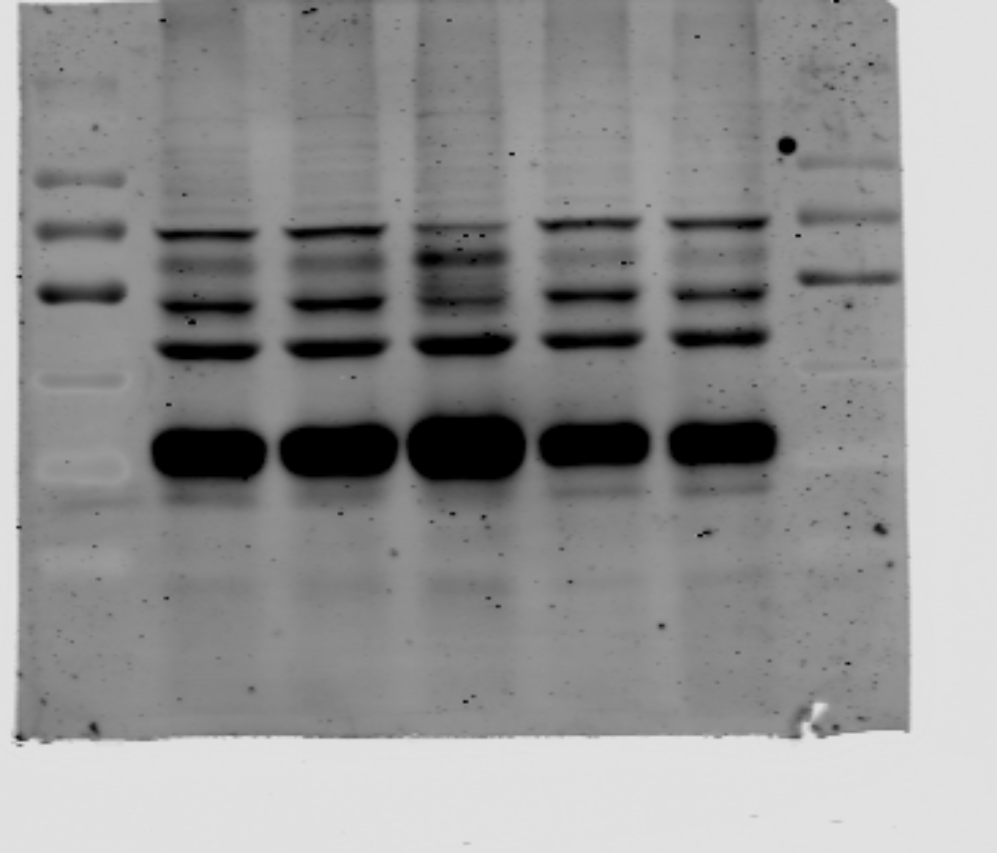

Supplement: Supplementary file 4 [file DataSheet2.zip › WB/Fig5/THP1-LPS+Niger+siANGPT/THP1-siANGPT1-ANGPT1.tif]

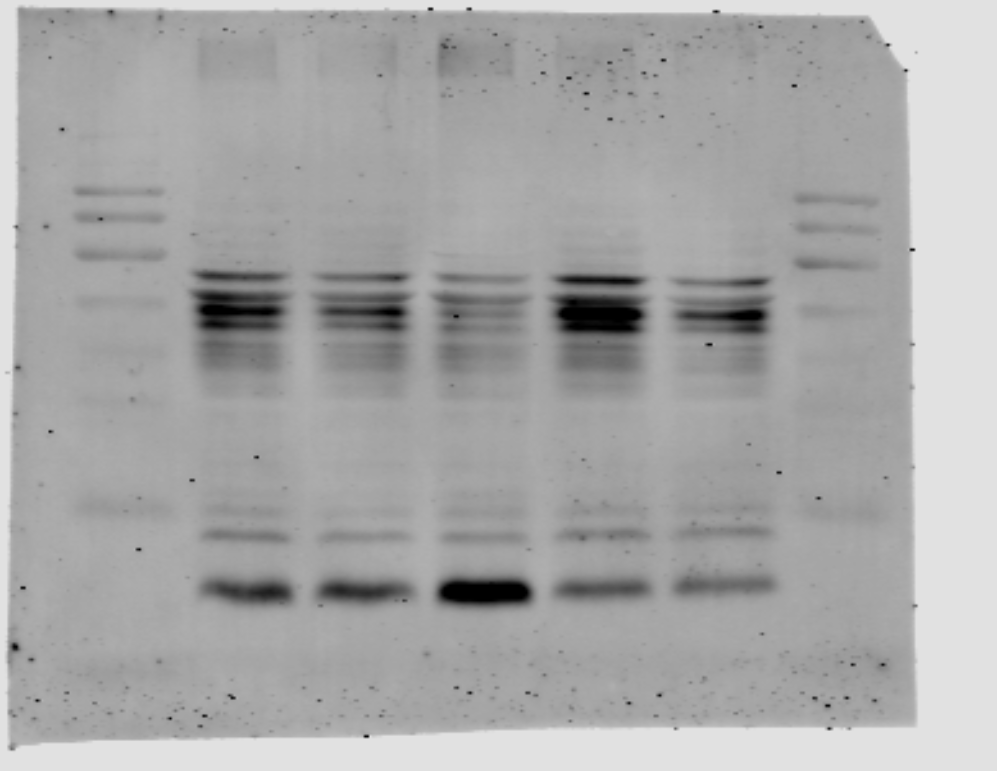

Supplement: Supplementary file 4 [file DataSheet2.zip › WB/Fig5/THP1-LPS+Niger+siANGPT/THP1-siANGPT1-Caspase1.tif]

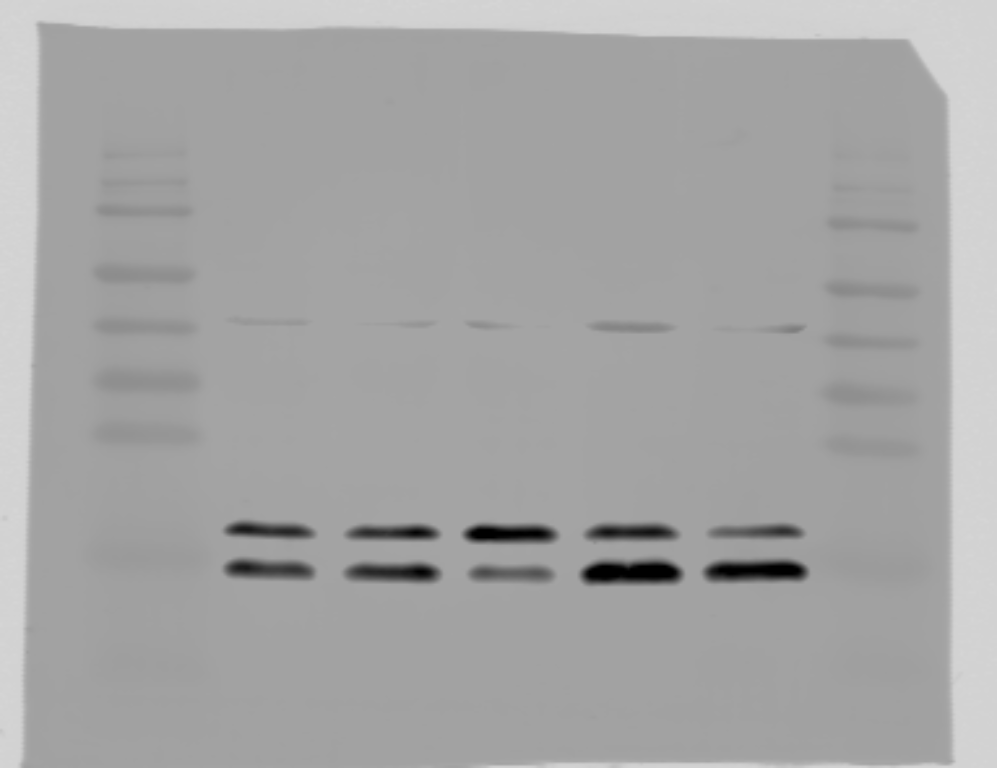

Supplement: Supplementary file 4 [file DataSheet2.zip › WB/Fig5/THP1-LPS+Niger+siANGPT/THP1-siANGPT1-GABARAP.tif]

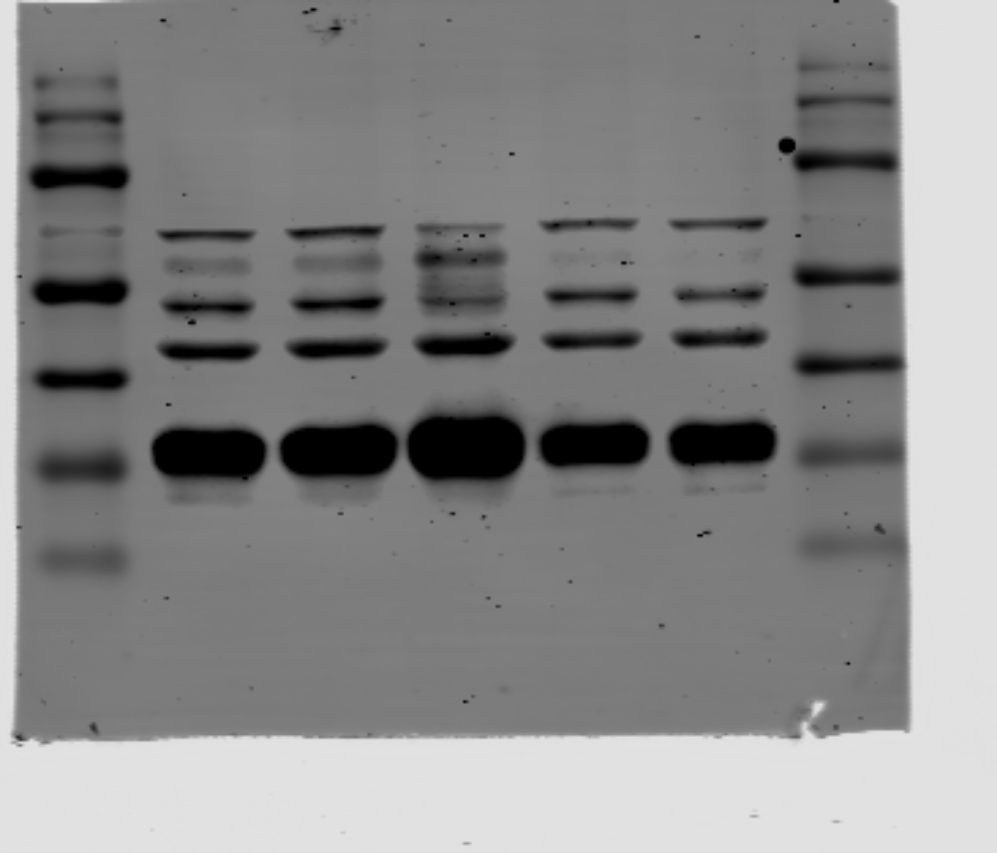

Supplement: Supplementary file 4 [file DataSheet2.zip › WB/Fig5/THP1-LPS+Niger+siANGPT/THP1-siANGPT1-GSDMD-actin.tif]

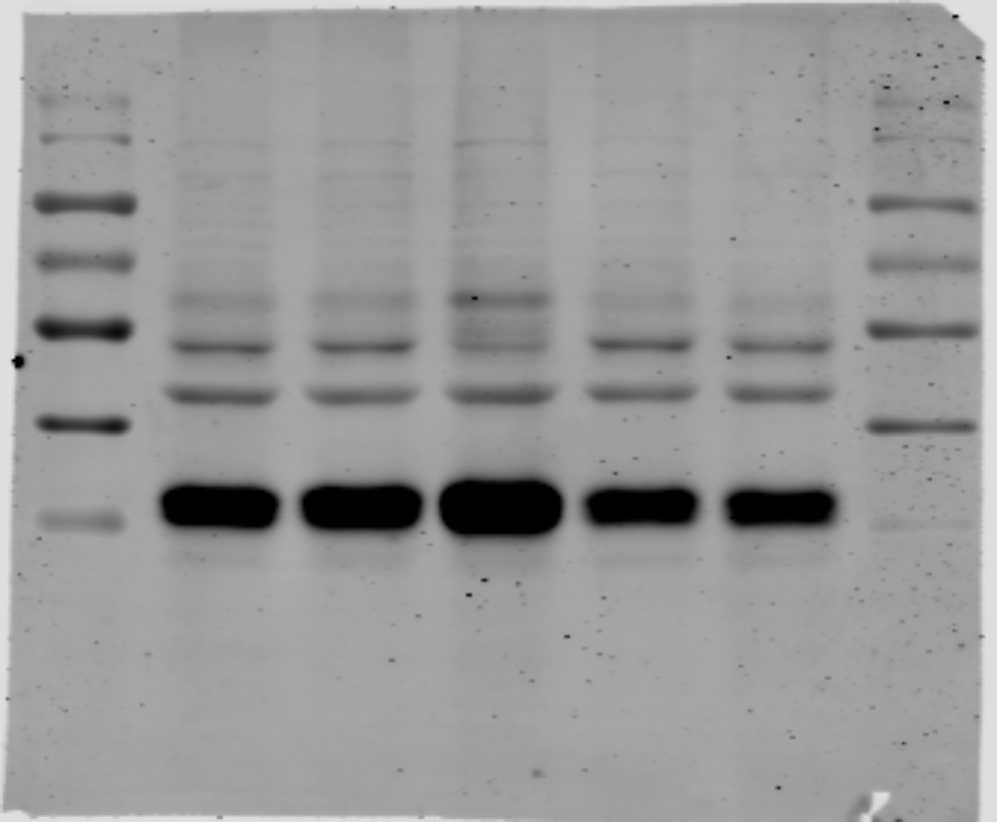

Supplement: Supplementary file 4 [file DataSheet2.zip › WB/Fig5/THP1-LPS+Niger+siANGPT/THP1-siANGPT1-GSDMD.tif]

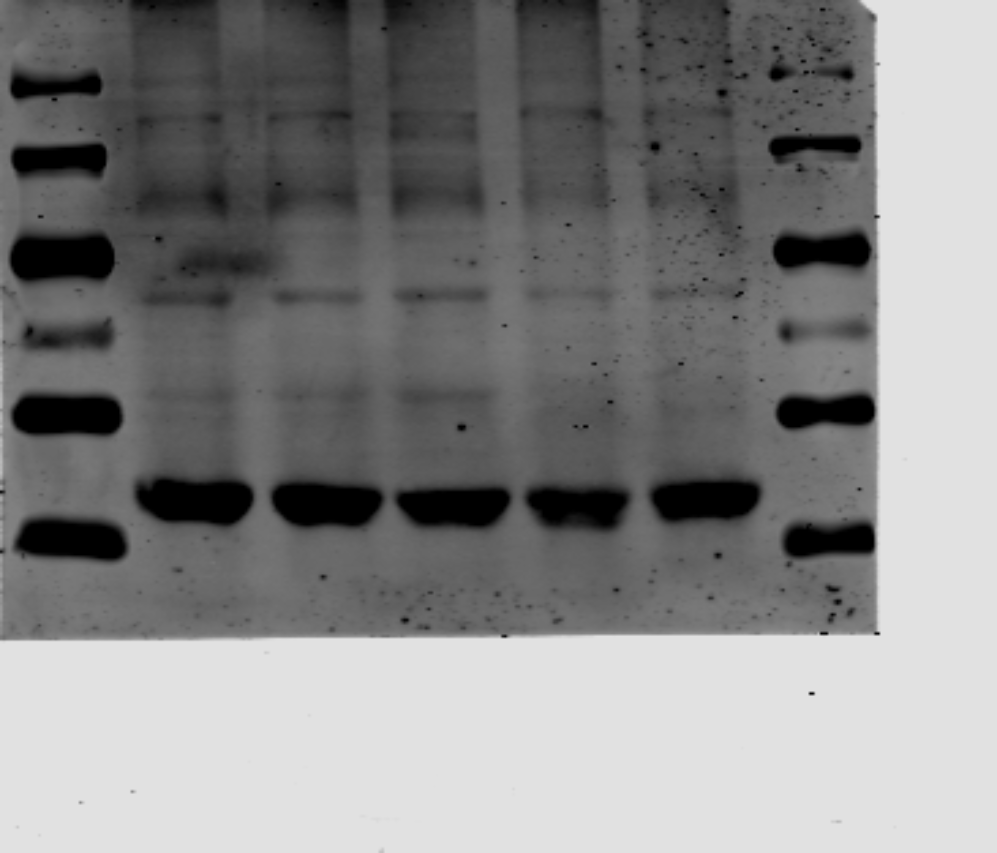

Supplement: Supplementary file 4 [file DataSheet2.zip › WB/Fig5/THP1-LPS+Niger+siANGPT/THP1-siANGPT1-NLRP3 - 副本.tif]

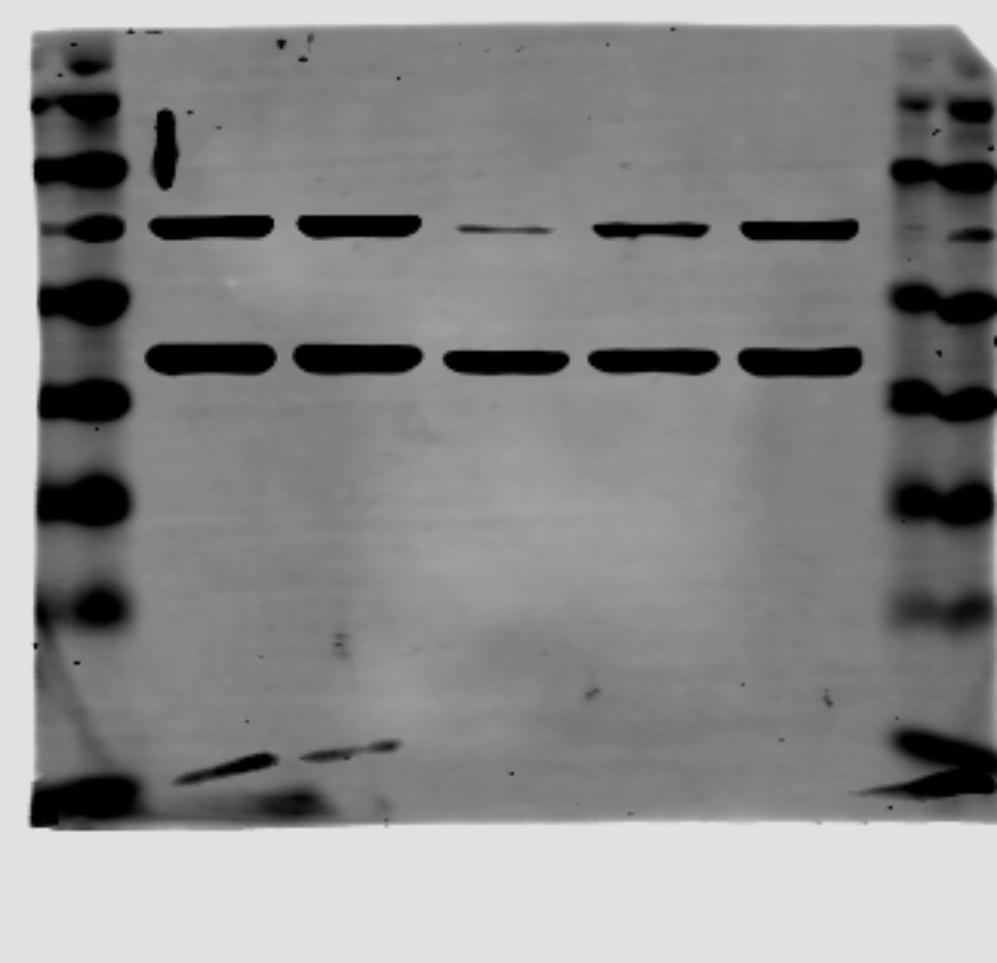

Supplement: Supplementary file 4 [file DataSheet2.zip › WB/FIgS2/NCM460 siANGPT-1/NCM460-siANGPT1.tif]

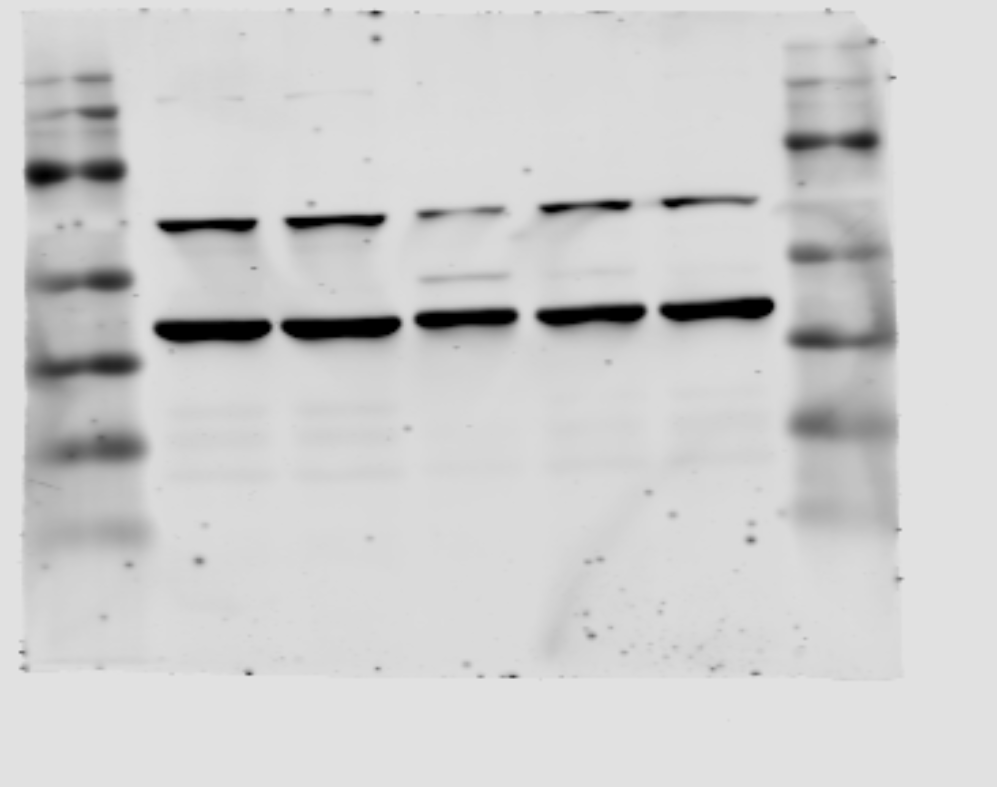

Supplement: Supplementary file 4 [file DataSheet2.zip › WB/FIgS2/THP1 siANGPT-1/THP-1-siANGPT1.tif]
